# *In Silico* Approach to Inhibition of Signaling Pathways of Toll-Like Receptors 2 and 4 by ST2L

**DOI:** 10.1371/journal.pone.0023989

**Published:** 2011-08-29

**Authors:** Shaherin Basith, Balachandran Manavalan, Rajiv Gandhi Govindaraj, Sangdun Choi

**Affiliations:** Department of Molecular Science and Technology, Ajou University, Suwon, Korea; University of South Florida College of Medicine, United States of America

## Abstract

Toll-like receptors (TLRs) activate a potent immunostimulatory response. There is clear evidence that overactivation of TLRs leads to infectious and inflammatory diseases. Recent biochemical studies have shown that the membrane-bound form of ST2 (ST2L), a member of the Toll-like/IL-1 receptor superfamily, negatively regulates MyD88-dependent TLR signaling pathways by sequestrating the adapters MyD88 and Mal (TIRAP). Specifically, ST2L attenuates the recruitment of Mal and MyD88 adapters to their receptors through its intracellular TIR domain. Thus, ST2L is a potent molecule that acts as a key regulator of endotoxin tolerance and modulates innate immunity. So far, the inhibitory mechanism of ST2L at the molecular level remains elusive. To develop a working hypothesis for the interactions between ST2L, TLRs (TLR1, 2, 4, and 6), and adapter molecules (MyD88 and Mal), we constructed three-dimensional models of the TIR domains of TLR4, 6, Mal, and ST2L based on homology modeling. Since the crystal structures of the TIR domains of TLR1, 2 as well as the NMR solution structure of MyD88 are known, we utilized these structures in our analysis. The TIR domains of TLR1, 2, 4, 6, MyD88, Mal and ST2L were subjected to molecular dynamics (MD) simulations in an explicit solvent environment. The refined structures obtained from the MD simulations were subsequently used in molecular docking studies to probe for potential sites of interactions. Through protein-protein docking analysis, models of the essential complexes involved in TLR2 and 4 signaling and ST2L inhibiting processes were developed. Our results suggest that ST2L may exert its inhibitory effect by blocking the molecular interface of Mal and MyD88 adapters mainly through its BB-loop region. Our predicted oligomeric signaling models may provide a basis for the understanding of the assembly process of TIR domain interactions, which has thus far proven to be difficult via *in viv*o studies.

## Introduction

Toll-like receptors (TLRs) are of interest to immunologists due to their front-line role in the initiation of innate immunity against invading pathogens [1]. TLRs play essential roles in the innate immune response to microbial pathogens based on their ability to recognize pathogen-associated molecular patterns (PAMPs) [2]. TLRs are type I transmembrane glycoproteins characterized by the presence of an extracellular domain (ectodomain, ECD) containing leucine-rich repeats (LRRs), which are primarily responsible for mediating ligand recognition, followed by a single transmembrane helix and an intracellular Toll-like/interleukin (IL)-1 receptor (TIR) domain, which is responsible for mediating downstream signaling [3]. So far, 10 and 12 functional TLRs have been identified in humans and mice, respectively, with TLR1-9 being conserved in both species. Mouse TLR10 is not functional due to retrovirus insertion, whereas TLR11, 12, and 13 have been lost from the human genome [4], [5]. The initial step in signal transduction involves dimerization of two receptor chains, which is induced by the binding of a specific ligand. Alternatively, in the case of TLR7, 8 and 9, the receptor may be present in the cell as a preformed yet inactive dimer, and ligand binding may cause reorientation of the TIR domains [6]. In either case, the TLR-TIR domain interaction serves as a nucleating act for recruitment of downstream signaling adapter proteins. All TLRs utilize the MyD88 signaling pathway with the exception of TLR3, which exclusively uses the TRIF pathway, to induce the expression of proinflammatory cytokine genes [7].

**Figure 1:**
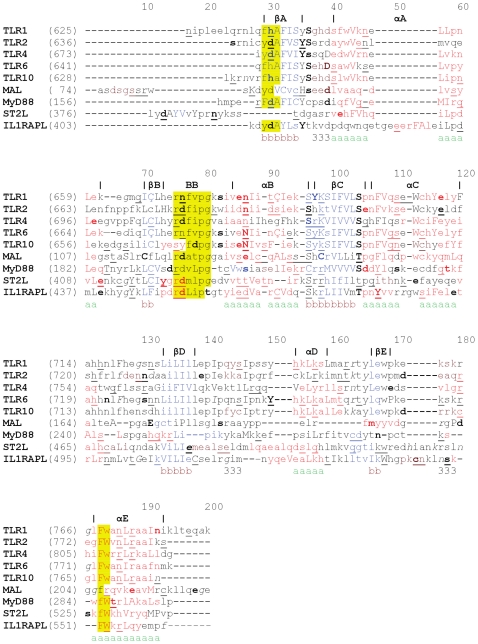
Structure-based sequence alignments of TIR domains. The JOY program was used to annotate the alignments for the TIR domains of TLR1, 2, 4, 6, 10, Mal, MyD88, ST2L and IL1RAPL. Numbers on top of alignment sequences are alignment positions. Three short sequence motifs (shaded in yellow color) called box 1–3 motifs are conserved among TIR domains. BB loop region of box 2 motif has been suggested to play a potent role in TIR-TIR interactions. Key to JOY annotations is as follows: solvent inaccessible - UPPER CASE; solvent accessible - lower case; α-helix - dark grey shaded; hydrogen bond to main chain amide **- bold**; hydrogen bond to main chain carbonyl - underline; positive φ - italic.

MyD88 (myeloid differentiation primary response gene 88), Mal (MyD88 adapter-like; also known as TIRAP, TIR domain-containing adapter protein), TRIF (TIR domain-containing adapter inducing IFN-β; also known as TICAM1, TIR domain-containing adapter molecule 1), TRAM (TRIF-related adapter molecule; also known as TICAM-2), and SARM (sterile α- and armadillo motif containing protein) are the five adapter proteins containing TIR domains that function in TLR signaling [5], [8]. These adapter proteins mediate TIR-TIR interactions between TLR receptors as well as receptor-adapter and adapter-adapter interactions, all of which are critical for signaling [9]. In general, the intracellular TIR domain of adapter proteins is composed of approximately 160 amino acid residues. The primary sequences of TIR domains are characterized by three conserved sequence boxes designated box 1, 2 and 3, as shown in Figure 1. Box 1 is considered to be the signature sequence of the family, whereas boxes 2 and 3 contain functionally important residues involved in signaling [10]. These processes result in the formation of a large multimer complex, or “signaling platform”, that propagates downstream signaling, eventually leading to the expression of several hundred primary immune response genes [11]. However, the architecture of the TLR signaling complexes is poorly understood currently due to a lack of reliable methods to study such interactions as well as the inherent weaknesses of individual inter- and intra-protein interactions in transitory complexes.

TLRs are double-edged swords, playing dual roles as physiological and pathological mediators. The dysfunction of TLRs has been implicated in a wide range of human diseases, including infectious diseases, immunodeficiency, sepsis syndromes, autoimmune disorders, artherosclerosis, malignancy and asthma [12]. As the immune system must constantly strike a balance between activation and inhibition mechanisms in order to avoid detrimental and inappropriate inflammatory responses, TLR signaling should therefore be under tight negative regulation. Although most members of the TLR/IL-1R superfamily are positive regulators of signaling, there are relatively fewer members that act as negative regulators in TLR signaling. So far, a number of negative regulators that suppress TLR signaling pathways at multiple levels have been identified [4].

ST2 is one of the potent negative regulators in TLR signaling. ST2 (also known as T1, Fit-1, or DER4) is present in two main forms (ST2L and sST2) and is encoded from a single ST2 gene by mRNA splicing [13], [14], [15]. ST2L is a type I transmembrane protein with three extracellular immunoglobulin-like domains and an intracellular TIR domain. sST2 (the soluble form) is identical to the extracellular region of ST2L except for an extra nine amino acids at the C-terminus [13], [14], [15]. ST2L is mainly expressed by cells of the major haematopoietic organs, whereas sST2 is present in both haematopoietic and non-haematopoietic cells [15]. ST2L is selectively expressed by TH2 cells, but not TH1 cells, and is associated with TH2-cell functions [16], [17], [18]. Although ST2L can drive activation of the MAPK (mitogen-activated protein kinase) pathway, it does not activate NF-κB [19], instead downregulating NF-κB activation in response to stimulation with IL-1 or LPS.

Macrophages from ST2-deficient mice (lacking both ST2L and sST2) produce markedly more proinflammatory cytokines in response to IL-1 and LPS, bacterial lipopeptides and CpG, but not to the TLR3 ligand poly I∶C [20], indicating that ST2L might directly affect the MyD88-dependent pathway. Consistent with this, overexpression of ST2L inhibits IL-1 receptor and TLR4-mediated but not TLR3-mediated NF-κB activation as well as attenuates the functions of MyD88 and Mal but not TRIF or IRAK [20]. Furthermore, ST2L co-precipitates with MyD88 and Mal but not TRIF or IRAK, and a mutant form of ST2L with a mutated Pro residue in box 2 of its TIR domain lacks suppressive activity. Together, these findings indicate that ST2L suppresses IL-1 and TLR signaling by sequestration of MyD88 and Mal through its TIR domain. However, ST2-deficient mice are no more susceptible to LPS shock than are wild-type mice, although they are unable to develop LPS tolerance under both *in vitro* and *in vivo* conditions [20]. This can be explained by the fact that ST2L is normally present intracellularly in resting cells and is only expressed on the cell surface after at least 4 hours of LPS stimulation. This amount of time lag might be too much for the control of septic shock, but it is sufficient to manifest endotoxin tolerance. Hence, ST2L performs an effective negative-feedback function in selective TLR signaling, including contributing to endotoxin tolerance and inhibiting TH1-cell responses. Although the significance of ST2L has been widely acknowledged, its inhibition mechanism remains unclear owing to a lack of structural information.

**Figure 2:**
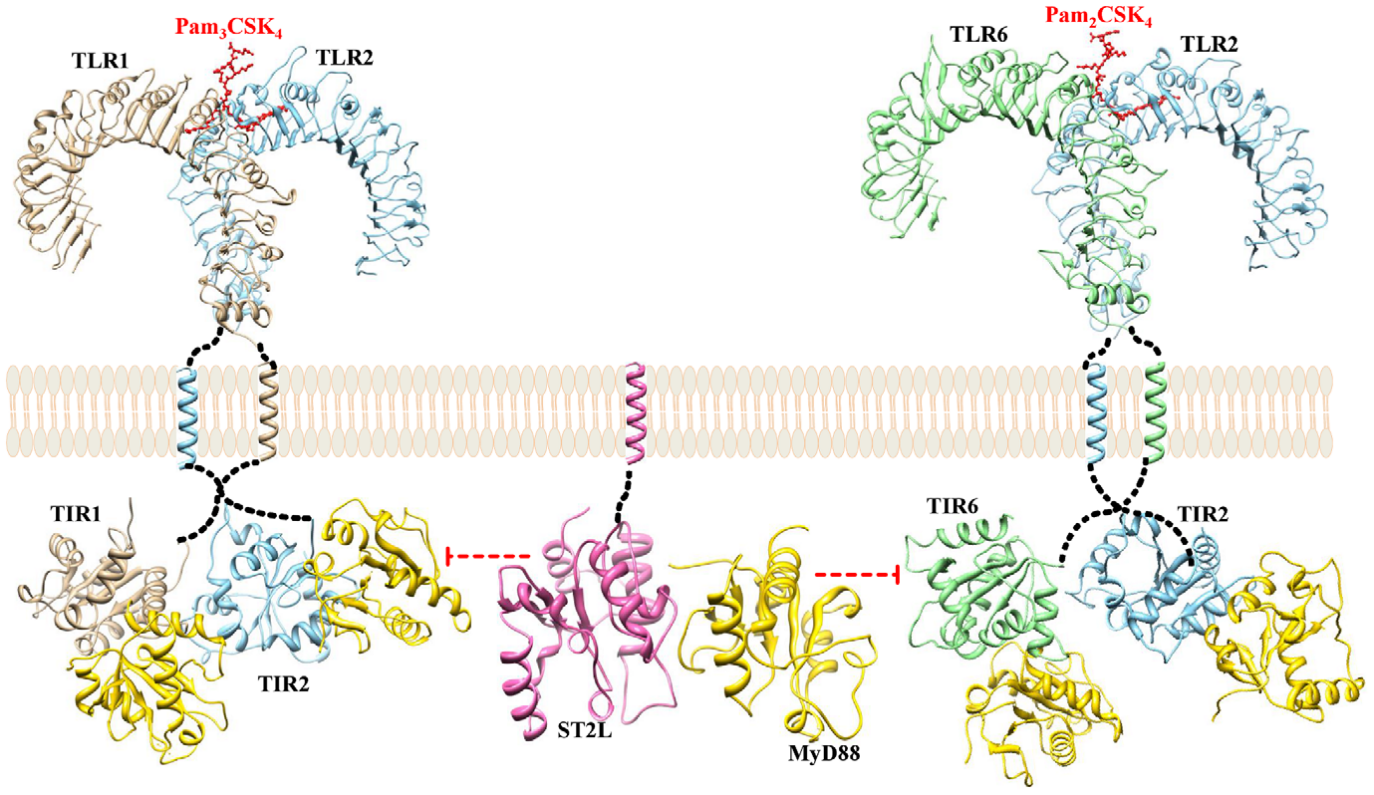
Models of ST2L inhibiting the TLR2 signaling pathway. Pictorial representation of ST2L inhibiting MyD88-dependent TLR2 (TLR2/1 and TLR2/6) signaling pathways. ST2L heterodimerizes with MyD88, thereby preventing the engagement of adapter protein MyD88 into the post-receptor signaling complexes (TLR2/1-MyD88 tetramer and TLR2/6-MyD88 tetramer) and thus exhibiting its inhibitory effect. Full-length structures of TLR2, 1 and 6 are colored in sky blue, tan and light green, respectively. The TIR domains of ST2L and MyD88 are colored in hot pink and gold, respectively. The available TLR2/1 and TLR2/6 ECD structure coordinates along with their respective ligands were taken from the PDB (2Z80 and 3A79). The red color dotted line along with the bar represents inhibition symbol.

**Figure 3:**
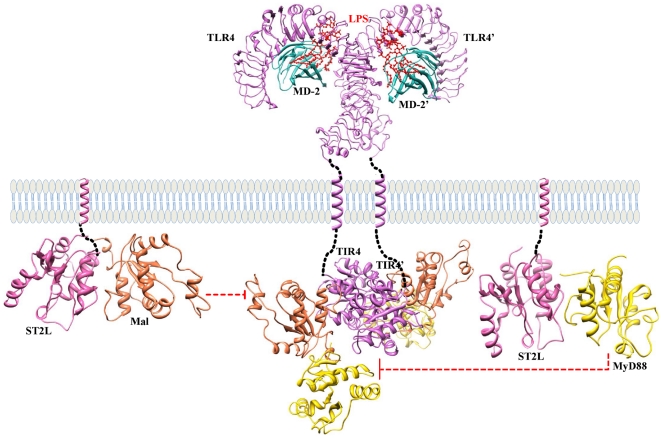
Models of ST2L inhibiting the TLR4 signaling pathway. Pictorial representation of ST2L inhibiting MyD88-dependent TLR4 signaling pathway. ST2L heterodimerizes with Mal and MyD88 by occupying their receptor-adapter and adapter-adapter interacting sites, thereby preventing the engagement of signaling adapters, Mal and MyD88, into the post-receptor signaling complex (TLR4-Mal-MyD88 hexamer) and thus exhibiting its inhibitory effect. Full-length structure of TLR4 is colored in orchid. The TIR domains of ST2L, Mal and MyD88 are colored in hot pink, coral and gold, respectively. The available TLR4 ECD structure coordinates along with its ligand was taken from the PDB (3FXI). The red color dotted line along with the bar represents inhibition symbol.

In this study, we used homology modeling techniques to construct three-dimensional models of the TIR domains of TLR4, 6, ST2L and Mal. So far, the crystal structures of the TIR domains of human TLR1, 2 as well as the NMR solution structure of MyD88 are known. All of the structures were subjected to MD simulation studies. Subsequently, the refined structures were used in protein-protein docking studies. Models of the essential complexes involved in TLR2 and 4 signaling and the ST2L inhibiting processes were proposed based on the results of the protein-protein docking studies in order to identify and quantify the residue-detailed structural inhibition framework (Figures 2 and 3).

## Materials and Methods

### Template Identification, Sequence Alignment, and Model Construction and Assessment

Amino acid sequences of the target proteins, human ST2L (BAA82405), TLR4 (O00206), TLR6 (BAA78631), and Mal (NP_683708), were obtained from the NCBI protein database [21]. Due to their high homology, crystal structures of the TIR domains of TLR1 (PDB ID: 1FYV), 2 (PDB ID: 1FYW) and 10 (PDB ID: 2J67; A) were used as common templates to build structural models of the TIR domains of TLR4, TLR6 and Mal. Due to the low sequence identity of ST2L with the three common templates, we used IL-1RAPL (PDB ID: 1T3G; A) as a single template to build the model of ST2L. Moreover, the overall structure of ST2L is similar to the structure of the Type I IL-1 receptor. To generate three-dimensional (3D) models of the TIR domains, we used a homology modeling approach as implemented in the program MODELLER version 9v3 [22]. Alignment of each target protein with the templates was generated using JOY (Figure 1). This program annotates protein sequence alignments with 3D structural features. Further, it helps to display 3D structural information in a sequence alignment in order to understand the conservation of amino acids in their specific local environments [23]. We used this alignment in MODELLER 9v3 to build 20 models for each target, of which the best model was selected based on stereochemical evaluations. The protein stereochemical quality was checked by employing ProQ [24], ModFOLD [25] and MetaMQAP [26].

### Molecular Dynamic Simulation

MD simulation studies were carried out as previously described [27]. MD simulations for all of the models were performed using YASARA dynamics [28] with AMBER03 force field under periodic boundary conditions following the explicit solvent method [29]. The modeled and solved structures of the TIR domains of TLR1, 2, 4, 6, Mal, MyD88 and ST2L were constructed around a complex with a 7.9 Å cutoff for Lennard-Jones forces and a direct space portion of electrostatic forces, which were calculated using the Particle Mesh Ewald method. The pKa values of the ionizable groups in the model were predicted and assigned protonation states based on pH 7.0. The cell was then filled with water, and the AMBER03 electrostatic potential was evaluated for all water molecules; the ones with the lowest or highest potential were turned into sodium or chloride counter ions, respectively, until the cell was neutral. Short steepest descent minimization of all atoms was performed to remove severe bumps in the protein. A start-up simulation was then carried out for 5 picoseconds (ps) using multiple time steps of 1.25 femtoseconds (fs) for intramolecular and 2 fs for intermolecular forces, with all heavy protein atoms fixed such that the solvent molecules smoothly covered the protein surface. Simulated annealing minimizations were carried out at 298 K, and the velocities were scaled down every 10 steps for a total time of 5 ps over 500 steps. All of the systems except ST2L were equilibrated for 2 nanoseconds (ns), whereas, ST2L was equilibrated for 4 ns. Finally, production run was carried out for 5.5 ns by storing the coordinates of all the atoms every 2.5 ps. The simulations were carried out using the AMBER03 force field at 298K and 0.9% NaCl [29]. Thus, the trajectory taken from these 5.5 ns simulations consisted of 2200 frames. However, the final snapshot obtained at the end of the simulations was considered to show a representative structure of all of the models that were further subjected to energy minimization and subsequently utilized for docking studies.

### Protein-Protein Docking

Unrestrained pairwise protein docking included seven TIR domain complexes: TLR4-TLR4 homodimer, TLR4-Mal tetramer, TLR4-Mal-MyD88 hexamer, TLR2/1-MyD88 tetramer, TLR2/6-MyD88 tetramer, ST2L-Mal and ST2L-MyD88. The refined structures were subjected to molecular docking studies. We used GRAMM-X [30] and ZDOCK [31], which are the most widely accepted rigid-body protein-protein docking programs, to predict and assess the interactions in the above-mentioned complexes. These two programs rank the 100 most probable predictions out of thousands of candidates based on the geometry, hydrophobicity and electrostatic complementarity of the molecular surface. The final docked complexes were selected from these top 100 predictions by implementing further qualifications, including (i) residue conservation of the interaction sites; (ii) N-terminal ends of the docked complexes should be oriented towards the cell membrane; and (iii) knowledge from previously published articles ([32], [33], [34], [35], [36] and [37]). This three-step filtering method resulted in a unique solution. The GRAMM-X/ZDOCK ranking of all optimal models is detailed in Table 1. The top-ranked complex present between the two docking programs, which are listed in Table 1, was considered as the final complex to be used for the identification of potential interacting residues across the interfaces. The buried surface interaction area of the docked models was calculated using the protein interfaces, surfaces and assemblies service (PISA) at the European Bioinformatics Institute [38]. The structural superimpositions and molecular electrostatics were calculated using Superpose v1.0 [39] and the nonlinear Poisson-Boltzmann equation with the APBS tools plugin for Pymol.

**Table 1:** Ranking of the selected docked complex.

COMPLEX	ZDOCK	GRAMM-X
TLR4 dimer	2	1
TLR4 dimer-Mal	7	5
TLR4-Mal tetramer	12	8
TLR4-Mal tetramer-MyD88	14	35
TLR4-Mal-MyD88 hexamer	21	39
TLR2/1 dimer-MyD88	6	32
TLR2/1-MyD88 tetramer	15	28
TLR2/6 dimer-MyD88	6	32
TLR2/6-MyD88 tetramer	26	37
ST2L-Mal	64	21
ST2L-MyD88	44	10

## Results

### Molecular Modeling of TLR4, 6, Mal and ST2L TIR Domains

In the secondary structure-aided alignments for homology modeling, the average target-template sequence identities of the TIR domains of TLR4, TLR6 and Mal with respect to multiple templates (TIR domains of TLR1, 2 and 10) were 40.69%, 86.81% and 21.80%, respectively. Additionally, the sequence identity of ST2L with the single template IL-1RAPL was 39.47%. The final modeled structures of the TIR domains all exhibited a typical TIR domain conformation, which is in agreement with the secondary structure prediction made by JPred [40]. TIR domains fold into a characteristic α/β structure with five-stranded parallel β-sheets surrounded by five α-helices on each side [41]. The loops that connect the secondary structure elements of the TIR domain are more variable, which may confer specificity for homo- and hetero-typic interactions between different TIR domains [35]. The JOY [23] output also showed that the residues in the models were in environments similar to those of the templates (Figure 1). Evaluation of the models involved analysis of the geometry, stereochemistry and energy distribution of the models. The evaluation listed in Table 2 indicates high quality in terms of overall packing for all of the models, which were subsequently used for MD simulation studies.

**Table 2:** Model evaluation of TIR domains.

MODEL	ProQ_LG/MX	ModFOLD_Q/P	MetaMQAP_GDT/RMSD
TLR1	6.055/0.370	0.5874/0.0249	83.540/1.598
TLR2	4.240/0.239	0.4512/0.0689	66.779/2.696
TLR4	5.608/0.362	0.6281/0.0212	82.877/1.791
TLR6	4.436/0.487	0.5984/0.0236	86.111/1.257
Mal	2.885/0.389	0.4639/0.0608	57.667/3.956
MyD88	7.319/0.518	0.5398/0.0343	82.624/1.784
ST2L	3.321/0.320	0.5287/0.0367	65.644/2.998

Note: ProQ_LG: >1.5 fairly good; >2.5 very good; >4 extremely good. ProQ_MX: >0.1 fairly good; >0.5 very good; >0.8 extremely good. ModFOLD_Q: >0.5 medium confidence; >0.75 high confidence. ModFOLD_P: <0.05 medium confidence; <0.01 high confidence. MetaMQAP_GDT/RMSD: an ideal model has a GDT score over 59 and a RMSD around 2.0 Å.

### Structure Refinement and Stability Evaluation

**Figure 4:**
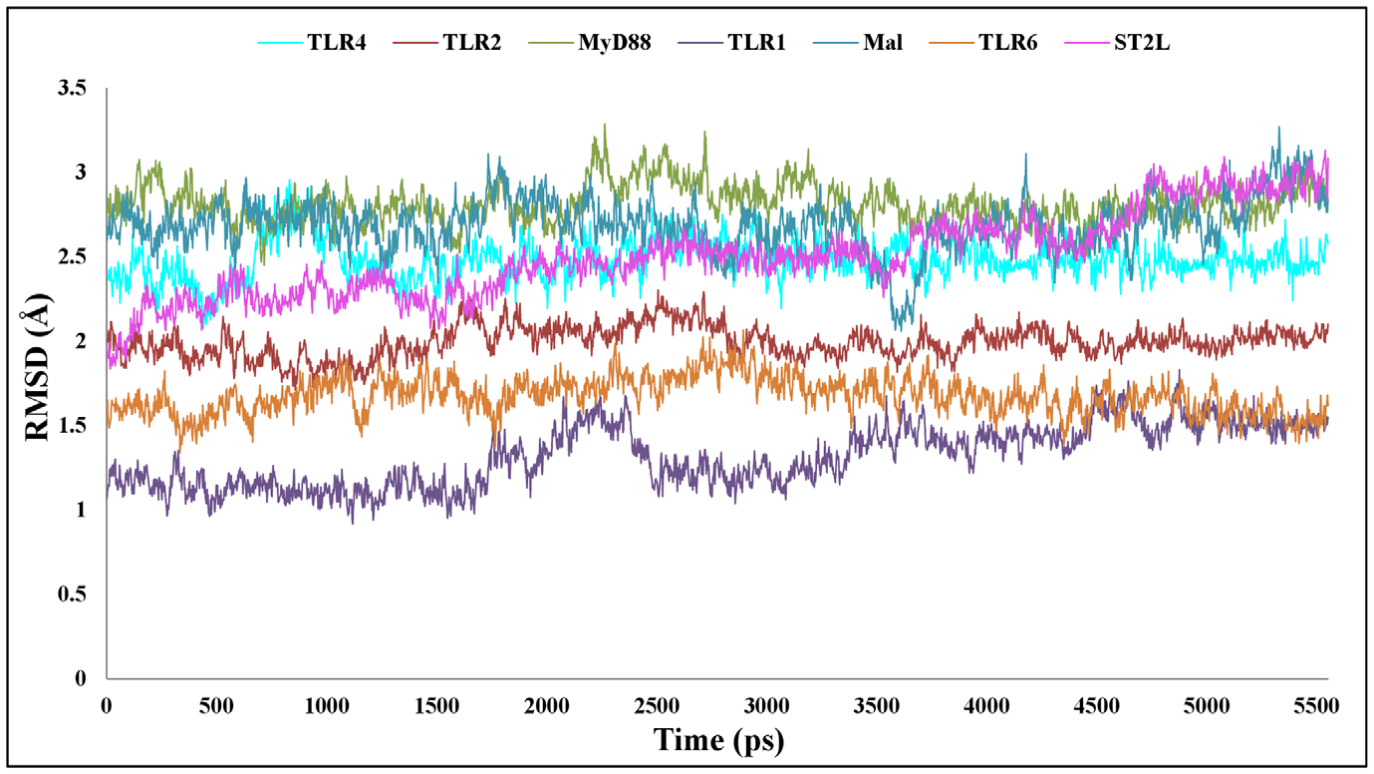
Molecular dynamic trajectory-based analysis of TIR domain model refinement. RMSD of Cα-atoms with respect to their initial structure shows the stable nature of the TIR domain model after the initial equilibration time.

**Figure 5:**
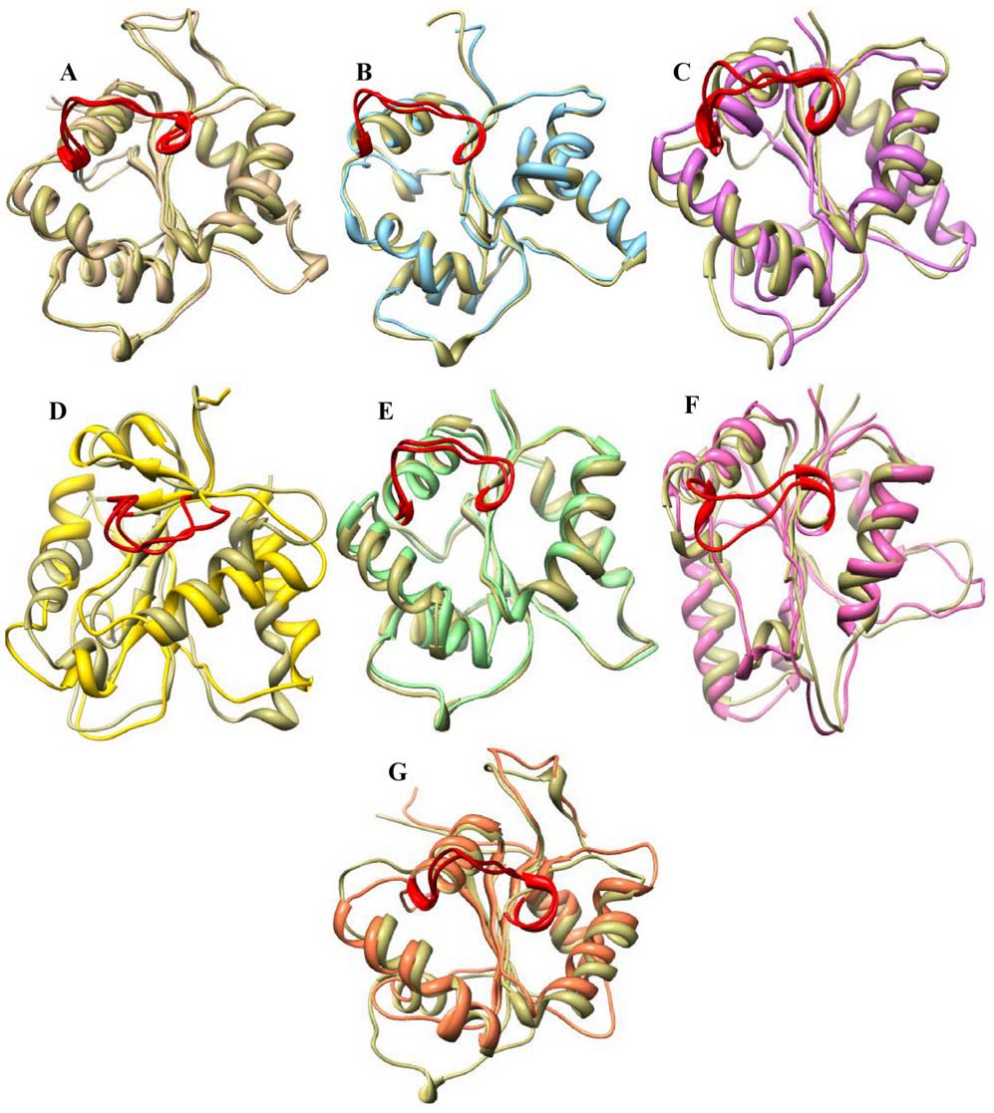
Superimposition of initial structure with the final snapshot obtained from MD simulation studies. Differences between the final snapshots of (A) TLR1 (tan), (B) TLR2 (sky blue), (C) TLR4 (orchid), (D) MyD88 (gold), (E) TLR6 (light green), (F) ST2L (hot pink) and (G) Mal (coral) and their respective initial structures are colored in khaki. Structural variations are mainly observed in the loop regions. TIR domains are shown with the BB loop colored in red facing the viewer.

The available TIR domain structure coordinates taken from the PDB (TLR1, TLR2 and MyD88) along with the constructed models (TLR4, TLR6, Mal and ST2L) were subjected to MD simulation in order to assess the stability of the models. Figure 4 shows the backbone RMSD plot for the protein Cα-atoms with reference to the initial structure and as a function of time. The plot shows that all of the models reached equilibrium state only after 2 ns of simulation and remained constant until the end of the dynamics, except in the case of ST2L, which reached an equilibrium state only after 4.5 ns of simulation. We then took the final snapshots of all the structures and subjected them to energy minimization. It is of worthwhile to note that these simulations are the longest explicit solvent MD simulations ever carried out on these TIR domains. Superimposition of the initial structure with the final refined structure of the TIR domains in each case (shown in Figure 5) revealed the following structural rearrangements: (i) within BB and CD loops and αD-helix region of TLR1 with a RMSD of 1.7 Å; (ii) within CD and DD loops of TLR2 with a RMSD of 1.8 Å; (iii) within all loop regions of TLR4 with a RMSD of 1.8 Å; (iv) within BB, CD and EE loop regions of TLR6 with a RMSD of 1.7 Å; (v) major variations within BB and CD loop regions and minor variations between other loops of Mal with a RMSD of 2.5 Å; (vi) within all loops and helices of MyD88 with a RMSD of 3.8 Å; (vii) and within all regions (helices, sheets and loops) of ST2L with a RMSD of 3.96 Å.

### Docking Benchmark

To assess the accuracy of the docking methods used in our study, we unrestrainedly inputted the TIR domain of human TLR10 (hTLR10), for which the dimeric crystal structure is already known as the most recent and well-accepted benchmark for TIR-TIR interactions. Docking experiments were carried out on the unbound structures (hTLR10 monomer), and the results were evaluated by comparison to the solved dimeric structure (PDB ID: 2J67). The native state of hTLR10 was present in the top 10 solutions of the two docking programs; it was ranked first by GRAMM-X and second ranked by ZDOCK. These results indicate that the docking methods we employed were reliable, and thus we utilized them in our subsequent docking studies.

### Pairwise Docking of TIR Domains

TIR domains are able to interact homo- or hetero-typically with each other. To demonstrate how ST2L inhibits the Mal/MyD88-dependent TLR2 and 4 signaling pathways, an understanding of the interaction mode of the TLR2 and 4 signaling complexes without ST2L is indispensible. The specificities of TIR-TIR interactions between adapters as well as between adapters and receptors define the formation of various complexes that initiate TLR signaling pathways. However, little is known about the mechanisms of heteromeric interactions between the TIR domains. We thus performed unrestrained rigid body docking for all of the TIR complexes, except the TLR2/6-MyD88 tetramer. The highest ranked model by GRAMM-X/ZDOCK was accepted as the optimal model, since it was ranked best on average. In all of the resulting docking models, we evaluated the interacting residues and interface surface areas. We also evaluated the experimentally validated amino acids and residues involved in interchain H-bonds as well as the charged residues present in the interfacial region, which are depicted in Tables 3, 4, 5, 6 and 7.

**Table 3:** List of interfacing residues between the TLR2/1 heterodimer complex and MyD88.

NO.	COMPLEX		INTERACTING RESIDUES
1	TLR2-MyD88	TLR2 (A)	*N638*, I639, C640, **Y641**, **D642**, E656, *N657*, **V660**, Q661, E664, N665, F666, *N667*, P668, P669, F670, *K671*, C673, R677, **D678**, I680, **P681**, *N688*, D691, K695, *K783*
		MyD88 (C)	C168, P169, S170, I172, S194, D195, **R196**, ***D197***, V198, S206, I207, A208, S209, *E210*, L211, K214, D226, Q229, S230, *E232*, *C233*, *F235*, K238, F239, *L241*, S242, L243, S244, P245
2	TLR1-MyD88	TLR1 (B)	**F637**, H638, F640, G645, S648, *K652*, C667, H669, *E670*, R671, ***N672***, F673, **P675**, G676, S678, I679, V680, E681, N682, I683, C686, Q703, S704, E705, *C707*, H708, L711
		MyD88 (D)	*R217*, R218, S244, *P245*, G246, *A247*, *K250*, F270, I271, T272, C274, R288, A292, S294, L295, P296
3	TLR2-TLR1	TLR2 (A)	K709, C713, *E716*, L717, F725, P746, *Q747*, *R748*, *C750*, K751, *R753*
		TLR1 (B)	Q632, N634, L635, *Q636*, F637, E660, *Q665*, *I666*, R671, N672, F673, V674, P675, N682, T685, C686, K689

Note: Biologically important residues are in bold, charged residues are underlined and residues involved in the formation of H-bond are in italic.

**Table 4:** List of interfacing residues between the TLR2/6 heterodimer complex and MyD88.

NO.	COMPLEX		INTERACTING RESIDUES
1	TLR2-MyD88	TLR2 (A)	S636, R637, *N638*, I639, C640, **Y641**, **D642**, E656, *N657*, **V660**, Q661, E664, N665, F666, *N667*, P668, P669, F670, *K671*, C673, R677, **D678**, I680, **P681**, K683, *N688*, D691, K695, *K783*
		MyD88 (C)	C166, Y167, C168, P169, S170, I172, S194, D195, **R196**, ***D197***, V198, S206, I207, A208, S209, *E210*, L211, K214, D226, Q229, S230, *E232*, *C233*, *F235*, K238, F239, *L241*, S242, L243, S244, P245
2	TLR6-MyD88	TLR6 (B)	F642, H643, Y648, *E650*, S653, K657, H674, *E675*, R676, *N677*, *F678*, V679, P680, G681, K682, S683, I684, V685, E686, *Q708*, S709, *E710*, C712, *H713*, L716
		MyD88 (D)	E213, *R217*, R218, *S244*, P245, G246, *A247*, H248, *Q249*, *K250*, *R251*, L252, I253, P254, F270, I271, T272, C274, R288, L289, A292, L293, L295, *P296*
3	TLR2-TLR6	TLR2 (A)	*E716*, L717, P746, *Q747*, R748, C750, K751
		TLR6 (B)	*Q641*, F642, *E665*, Q670, *I671*, R676, N677, V679, N690, C691, E693, K694

Note: Biologically important residues are in bold, charged residues are underlined and residues involved in the formation of H-bond are in italic.

**Table 5:** List of interfacing residues in the TLR4-Mal-MyD88 hexamer complex.

NO.	COMPLEX		INTERACTING RESIDUES
1	TLR4 dimer	TLR4	Y680, S681, *S682*, Q683, E685, R689, H708, Y709, F712, **P714**, **G715**, **V716**, A717, **I718**, A719, A720, N721, Q739, H740, F741, Q743, *S744*, R745, C747, I748, *Y751*, *E752*, A754, Q755
2	TLR4-Mal	TLR4 (A)	I713, G715, V716, A717, A719, A720, H724, *E725*, H728, K729, Y751, E752, I753, *A754*, Q755, T756, W757, Q758, F759, L760, *S761*, S762
		Mal (C)	*R81*, W82, *S83*, K84, D85, T124, **P125**, G126, G127, *A128*, V130, S131, E132, C134, *Q135*, S138, S139, L165, T166, E167, A168, P169, E221, G222, E223
3	Mal-MyD88	Mal (C)	A74, S75, D76, S77, G78, K84, D85, **Y86**, D87, V104, S105, E108, G109, S110, T111, A112, S113, L114, H141, R215, *K217*
		MyD88 (E)	H156, M157, P158, *E159*, R160, K190, S194, D195, **R196**, **D197**, W205, S206, I207, A208, R215
4	TLR4-Mal	TLR4 (B)	S682, *Q683*, E685, D686, R689, Q739, H740, Q743, *Y751*, Q772, T777
		Mal (C)	S83, D85, **Y86**, L120, R121, D122, A123, T124, P125, E132, *Q135*
5	TLR4-MyD88	TLR4 (A)	Q739, K773, E775, Y794, *E796*, S800
		MyD88 (E)	H156, R180, E183, *R188*, K190

Note: Biologically important residues are in bold, charged residues are underlined and residues involved in the formation of H-bond are in italic.

**Table 6:** List of interfacing residues between the ST2L-Mal complex.

NO.	COMPLEX		INTERACTING RESIDUES
1	ST2L-Mal	ST2L	L376, Y377, H402, **P406**, D407, E410, N411, K412, C413, *G414*, Y415, T416, L417, G421, *R422*, D423, M424, L425, P426, T433, T436, *N437*, K440, R532, P538
		Mal	A74, *S75*, S77, G78, S80, R81, S83, K84, *D85*, **Y86**, L114, R115, F117, L120, *R121*, D122, A123, T124, **P125**, Q135, H141

Note: Biologically important residues are in bold, charged residues are underlined and residues involved in the formation of H-bond are in italic.

**Table 7:** List of interfacing residues between ST2L-MyD88 complex.

NO.	COMPLEX		INTERACTING RESIDUES
1	ST2L-MyD88	ST2L	P384, *R385*, *N386*, Y387, K388, E398, H402, *Q403*, **P406**, D407, *E410*, N411, Y420, *G421*, R422, D423, M424, L425, P426
		MyD88	*H156*, *M157*, Q176, R180, E183, Q184, D195, ***R196***, ***D197***, V198, L199, **P200**, G201, T202, C203, W205, S206

Note: Biologically important residues are in bold, charged residues are underlined and residues involved in the formation of H-bond are in italic.

#### TLR4-TLR4 homodimer

A key concept in TLR signaling is stimulus-induced dimerization of the receptor ECDs, which causes a conformational rearrangement that is transmitted across the membrane, resulting in reorientation or homodimerization of the receptor TIR domains [42]. The TLR4 dimer complex obtained from the docking solutions is axially symmetric, similar to the dimeric crystal structure of hTLR10 [43]. It has an extensive and highly attuned interaction surface area. The TLR4 dimer displays two-fold symmetry with a buried surface interaction area of 967 Å^2^, which is in the typical range of physiological interaction surfaces. The surface area buried at the dimer interface of the TLR4 homodimer has contributions of 966 Å^2^ and 969 Å^2^ from each of the two protomers. Out of 146 residues, 30 residues are found in the interface region from each TLR4 monomer chain.

Major contributions to the dimer interface are made by residues of the BB-loops and the αC-helices. The dimer interface is made up of a hydrophobic core surrounded by H-bond network. The hydrophobic core is mainly constituted of six residues of the BB-loop (Y709, F712, P714, G715, V716 and A717). Apart from contributing to the dimer contact, these residues also play a significant role in stabilizing the observed conformation of the BB loop, consisting of residues L707-A717. Four residues (C747, I748, Y751 and A754) from the αC-helix, one residue (Y680) from the AA-loop, and three residues (I718, A719 and A720) from the αB-helix also contribute to the hydrophobic interactions. Ten hydrogen bonds are present in the dimer interface (Figure S1A). This includes two and three hydrogen bonds among TLR4 chains A and B. TLR4 chain A S682 forms two hydrogen bonds with TLR4 chain B residues A717 and A720. Likewise, TLR4 chain B S682 forms two hydrogen bonds with TLR4 chain A residues A717 and A720. Additionally, TLR4 chain A Y751 forms three hydrogen bonds with TLR4 chain B residues Q739, H740 and F741, thereby forming a Y-Q-Y-H-Y-F chain of hydrogen bonds. Similarly, TLR4 chain B residue Y751 forms three hydrogen bonds with TLR4 chain A residues Q739, H740 and F741, thereby connecting the two αC-helices at the very center of the dimer. The hydrophilic residues of TLR4 chain A form hydrogen bonds and ionic interactions with TLR4 chain B, which surrounds and supports the hydrophobic core of the dimerization interface. Our TLR4 dimer model is consistent with the mutagenesis study reported by Ronni et al [44].

#### TLR4-Mal tetramer

Cytoplasmic adapter proteins couple ligand-receptor interactions to intracellular signaling events. The TLR4-docked homodimer complex creates two specific symmetry-related binding sites at the homodimer interface to facilitate the binding of downstream signaling adapter proteins. Previous *in vitro* binding experiments demonstrated that MyD88-TIR does not directly bind to the cytosolic TIR domain of TLR4, whereas Mal-TIR does [45]. Of the MyD88-dependent pathways involving TLR2, 4, 5, 7 and 9, only the TLR2 and 4 pathways require Mal for efficient signal transduction, indicating a role for Mal as a bridging adapter.

**Figure 6:**
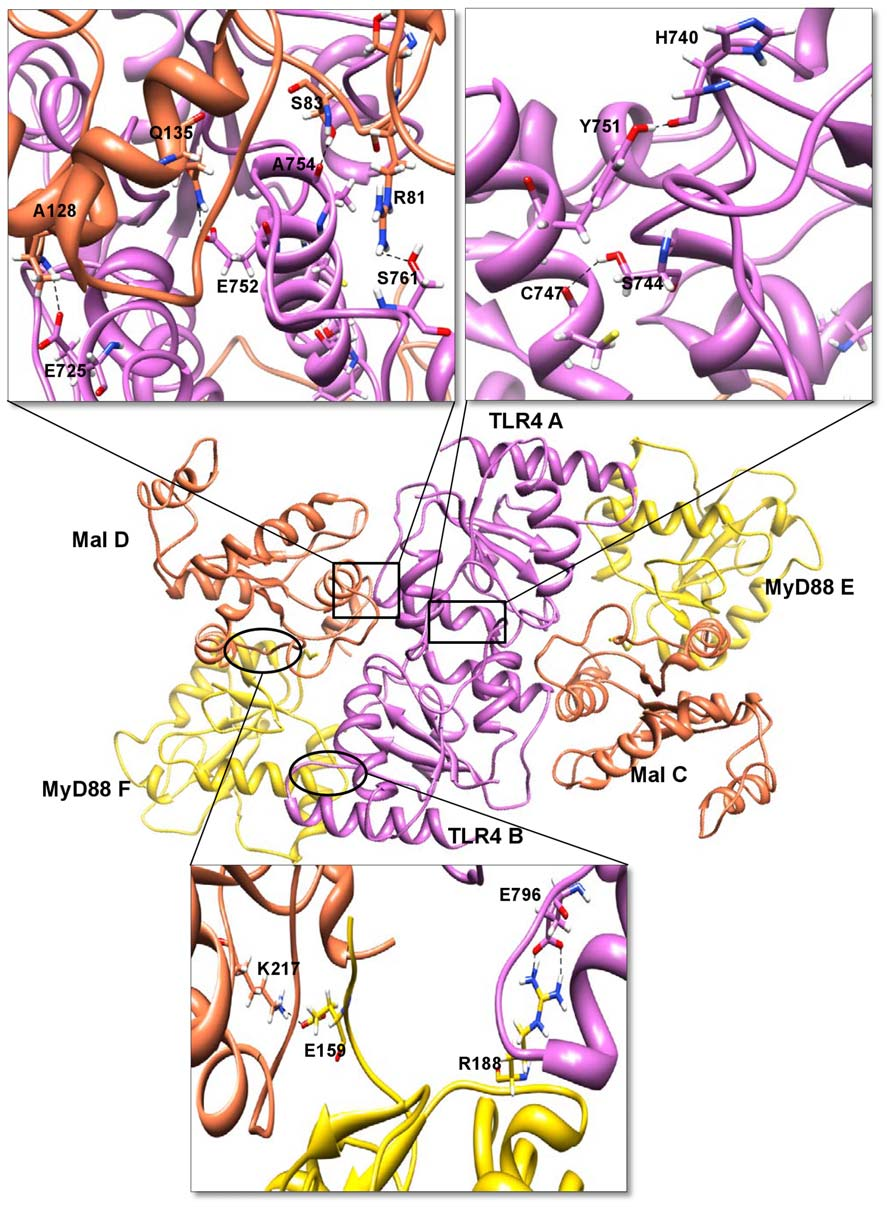
Interactions in the TLR4-Mal-MyD88 hexamer interface region. The TIR domains of TLR4, Mal and MyD88 are represented as ribbon models. TLR4 receptor chains A and B are shown in orchid color. Mal (C and D) and MyD88 (E and F) adapter chains are shown in coral and gold colors, respectively. The important structural motifs that contribute to the hexamer interface include BB-loop and αC-helices of TLR4, BB-loop and αB-helices of Mal and BB-loop of MyD88. The interacting residues in the hexamer residual interface region are highlighted in boxes. Side chains of the amino acids contributing to hydrogen bond formation are represented as a stick model with the residue names and numbers shown next to them. Black dotted lines represent the hydrogen bonds.

The 200 docking solutions provided by GRAMM-X and Z-Dock for Mal are located at the TLR4 dimer interface, indicating that the dimer formation presents two specific scaffolds for binding of the adapter molecules. The interaction surfaces provided by the TLR4 dimer interface are at either side of the structure rather than at the top, since that region would be sterically hindered by the membrane. Docking studies were carried out using the TLR4 dimer complex and Mal monomer. Two TLR4 dimer-Mal docked complexes with similar geometries but opposite orientations (flipped 180° to each other) were extracted from the docked solutions. Since two specific symmetric scaffolds are provided at the TLR4 homodimer interface, we superimposed these two docked complexes to obtain the final receptor-adapter tetrameric complex (TLR4-Mal tetramer – Figure 6).

The buried surface at the interface of the TLR4 dimer-Mal complex constitutes 1154 Å^2^ from the TLR4 dimer and 1161 Å^2^ from Mal. There are 10 residues from TLR4 chain A and 23 residues from chain B that make contact with 37 residues from Mal chain C. Similarly, 23 residues from TLR4 chain A and 10 residues from chain B make contact with 37 residues from Mal chain D. Previous studies have shown that P125H mutation of Mal results in decreased interactions between Mal and TLR4. However, this mutation does not have any effect on the interactions between Mal and MyD88 [46]. In line with these observations, our docked complex also shows that Mal P125 is at the interface region between TLR4 and Mal, thereby demonstrating a potent role for this residue in receptor-adapter interactions. At the interface of the TLR4 dimer-Mal complex, eight hydrogen bonds are present, donated by seven residues from each TLR4 chain and six residues from each Mal chain (Figure S1B). Two salt bridges are formed between TLR4 H724 (chains A and B) and Mal E132 (chains C and D) as well as TLR4 H740 (chains A and B) and Mal D85 (chains C and D). Additionally, another important ionic interaction between TLR4 H740 (chains A and B) and Mal D85 (chains C and D) has been observed.

The major contributions to the tetramer interface are made by residues of the BB-loops and αC-helices of TLR4 as well as by the residues of the BB-loops and αB-helices of Mal. Strong hydrophobic interactions are observed between hydrophobic residues from both components of the receptor-adapter complex. Apart from contributing to the receptor-adapter interaction, these hydrophobic residues also play a significant role in stabilizing the observed conformation of the TLR4 and Mal BB loops. Moreover, strong electrostatic interactions are present between charged residues from both components of the complex. The interface region of Mal is composed of eight negatively charged and three positively charged residues (Figure S2B, highlighted in blue dotted circles). In the case of the TLR4 dimer complex, four negatively and five positively charged residues are exposed (Figure S2A, highlighted in blue dotted circles). These data suggest that the predominant interactions between Mal and the TLR4 dimer are based on electrostatic interactions.

#### TLR4-Mal-MyD88 hexamer

MyD88 is a universal, cytosolic adapter protein composed of an N-terminal death domain (DD), C-terminal TIR domain, and a short connecting linker. The TIR domain of MyD88 has pivotal functions in the formation of signal initiation complexes involving the cytosolic domains of TLRs. MyD88 has been reported to be involved in signaling pathways initiated by all TLRs, with the exception of TLR3 and late signaling by TLR4 [1]. MyD88 serves as an essential “signaling adapter” that transmits signals from ligand-activated TLRs to downstream factors to initiate kinase-dependent signaling cascades, whereas Mal functions as a “sorting adapter” that recruits MyD88 to the plasma membrane via its PIP2-binding domain. Recently, Ohnishi et al. revealed the solution structure of MyD88-TIR using NMR spectroscopy (PDB ID: 2Z5V) [34]. We have used the same NMR solution structure of MyD88-TIR in our studies. Although full-length MyD88 forms a dimer, the isolated TIR domain was shown to exist as a monomer in solution state, which appears to be mediated via homomeric interactions within its death domain. Therefore, the reported MyD88 dimerization is likely mediated by DD+ID and not by the TIR domain.

Docking studies were carried out using the TLR4-Mal tetramer complex and MyD88 monomer. Similar docking procedure of the TLR4-Mal tetramer complex was employed to obtain the final hexameric complex (TLR4-Mal-MyD88), as shown in Figure 6. The buried surface at the interface of the TLR4-Mal-MyD88 hexameric complex constitutes 649 Å^2^ from the TLR4 dimer-Mal C complex and 658 Å^2^ from MyD88. In the docked complex, six residues from TLR4 chain A and 21 residues from Mal chain C make contact with 20 residues from MyD88 chain F. Similarly, six residues from TLR4 chain B and 21 residues from Mal chain D make contact with 20 residues from MyD88 chain E. Previous reports showed that Y86 mutation of Mal significantly alters the affinity of Mal for MyD88 [34], [47]. Furthermore, another recent study showed the importance of the R196 residue via mutation to cysteine in new primary immune-deficiency (MyD88 deficiency) patients. This mutation resulted in a significant decrease in the direct interaction between MyD88-TIR and Mal-TIR [34], [48]. These observations are consistent with our docked hexameric model, wherein Y86, R196 and D197 are present in the adapter-adapter molecular interface, further supporting the validity of our hexamer model.

It is interesting to note that binding of Mal does not induce any conformational changes in the TLR4 TIR dimer (receptor). However, binding of MyD88 induces conformational changes in both the receptor-receptor and receptor-adapter complexes, as evidenced by altered interactions (such as interfacing residues, hydrogen bonds (Figure S1C), salt bridges and buried surface area) in the final hexamer complex, which is in agreement with previous reports, thereby strengthening our final docking model. In the hexamer complex, two hydrogen bonds are present in the TLR4 dimer interface (AB). Four hydrogen bonds are present at the interface of the TLR4-Mal (AC) complex. One salt bridge is formed between TLR4 H724 (chain A) and Mal E132 (chain C). Since the hexamer is symmetrical, the same type of hydrogen bonds and salt bridge are formed in the other TLR4-Mal (BD) complex. A single hydrogen bond is present among TLR4 chains A and B and Mal chains D and C, respectively. Similarly a salt bridge is formed among TLR4 chains A and B (H740) and Mal chains D and C (D85). Residue K217 of Mal chains C and D forms both a hydrogen bond and salt bridge with E159 of MyD88 chains E and F, thus stabilizing the adapter-adapter interactions. There is a double hydrogen bond formed among TLR4 chains A and B and MyD88 chains E and F, respectively. A single salt bridge is formed among TLR4 E796 (chains A and B) and MyD88 R188 (chains E and F). The residues that participate in salt bridge formation also contribute to ionic interactions in the hexamer interface. Moreover, the predominant interactions in this hexamer complex are based on electrostatic interactions. Electrostatic potential studies have shown that the two basic patches present in the MyD88 molecule (Figure S2D, highlighted in blue dotted circles) interact largely with the negatively charged surface of the Mal molecule (Figure S2C, highlighted in blue dotted circles). However, there is also a slight interaction of the MyD88 basic patch with the negatively charged surface of TLR4 (Figure S2C, highlighted in blue dotted circles). The major contributions to the hexamer interface are made by the BB-loop residues and αC-helices of TLR4, BB-loop residues and αB-helices of Mal, and BB-loop residues of MyD88. Remarkably, both the modeling and electrostatic studies predict that the ‘BB’ loop structures of all three molecules (TLR4, Mal and MyD88) are critical determinants of binding specificity for receptor-receptor, receptor-adapter and adapter-adapter interactions.

#### TLR2-1/6 heterodimer

TLR2 is believed to function as a heterodimer, possibly with TLR1 or 6, in the recognition of foreign pathogens [49]. Tao et al. reported the crystal structure of the TIR domain of human TLR2 homomultimers (PDB ID: 1O77) [50]. This study suggested that the DD loop and BB loop may form points of contact between two molecules and that the asymmetric AB dimer may reflect the natural heterodimeric TLR2∶TLRx signaling complex, where x corresponds to TLR1 or 6. Furthermore, another group utilized computational docking models to guide alanine-scanning mutagenesis and demonstrated that the DD-loop region of TLR2 and BB-loop region of TLR1 participate in TLR2/1 heterodimerization [51]. Based on these previous studies [50], [51], we hypothesized that the DD loop of TLR2 might interact with the BB loop region of TLR1 or 6.

The refined crystal structures of the TIR domains of TLR1 (PDB ID: 1FYV) and 2 (PDB ID: 1FYW) were used in our studies. Since the crystal structure of the observed asymmetric AB dimer of human TLR2 TIR domain is consistent with several biological observations, we focused on this same asymmetric AB dimer in our analysis. We superimposed the refined structure of TLR1 with molecule B of the asymmetric AB dimer as well as the refined structure of TLR2 with molecule A, producing the final TLR2/1 heterodimer complex. The same procedure was followed to produce the final TLR2/6 heterodimer complex. These two complexes (TLR1-2 and TLR2-6) were energy minimized using AMBER03 force field and subsequently subjected to protein-protein docking studies to probe for the interaction sites of MyD88.

The surface area buried at the dimer interface of the TLR2/1 heterodimer has contributions of 490 Å^2^ and 499 Å^2^ from each of the two protomers. Likewise, TLR2/6 heterodimer has contributions of 338 Å^2^ and 293 Å^2^ from each of the two protomers. The interactions between these two TLR2 heterodimer structures (TLR2/1 and TLR2/6) mainly involve the BB loops of TLR1 or 6 and DD loop of TLR2. A large network of hydrogen bonds (shown in Figure S3A and B) present in the two heterodimer complexes (TLR2/1 and TLR2/6) mediates these heterodimer interfaces.

A recent study by Kenny et al. [52] demonstrated that TLR2 heterodimerizes with TLR1 or 6 to allow for Mal-independent recruitment of MyD88. At high ligand concentrations, TLR2 activation can occur in the absence of Mal due to greater coupling of TLR1 or 6 to the complex, which allows for sufficient MyD88 recruitment. On the other hand, at low ligand concentrations, this coupling might be less effective and therefore require Mal to stabilize MyD88 in the complex. Therefore, direct interactions between the TIR domains of MyD88 and TLR2-1/6 may mediate signal transduction as discussed before. We also extended the same hypothesis to obtain the receptor-adapter docked complex of TLR2 signaling (Mal-independent recruitment of MyD88). Our model of the receptor dimer (TLR2-1/6) docking to the adapter (MyD88) provides additional residual information on TLR2 signaling.

#### TLR2/1-MyD88 tetramer

Previous studies have shown that the conserved Pro in box 2 of the TIR domain (TLR1 P675 or TLR2 P681) is necessary for signaling. Moreover, P681 mutation abrogates the TLR2 TIR domain-MyD88 interaction [45], [51]. Based on these previous reports, we hypothesized that the BB loop side of the TLR1 and 2 TIR domains might interact with MyD88. To test our hypothesis, we performed computer-assisted docking studies of the TLR2/1-MyD88 complex. We filtered out the final TLR2/1-MyD88 docked complex based on our hypothesis. The docking solutions provided by the docking programs for MyD88 are located at the sides of the TLR2/1 heterodimer interface (BB loop region).

**Figure 7:**
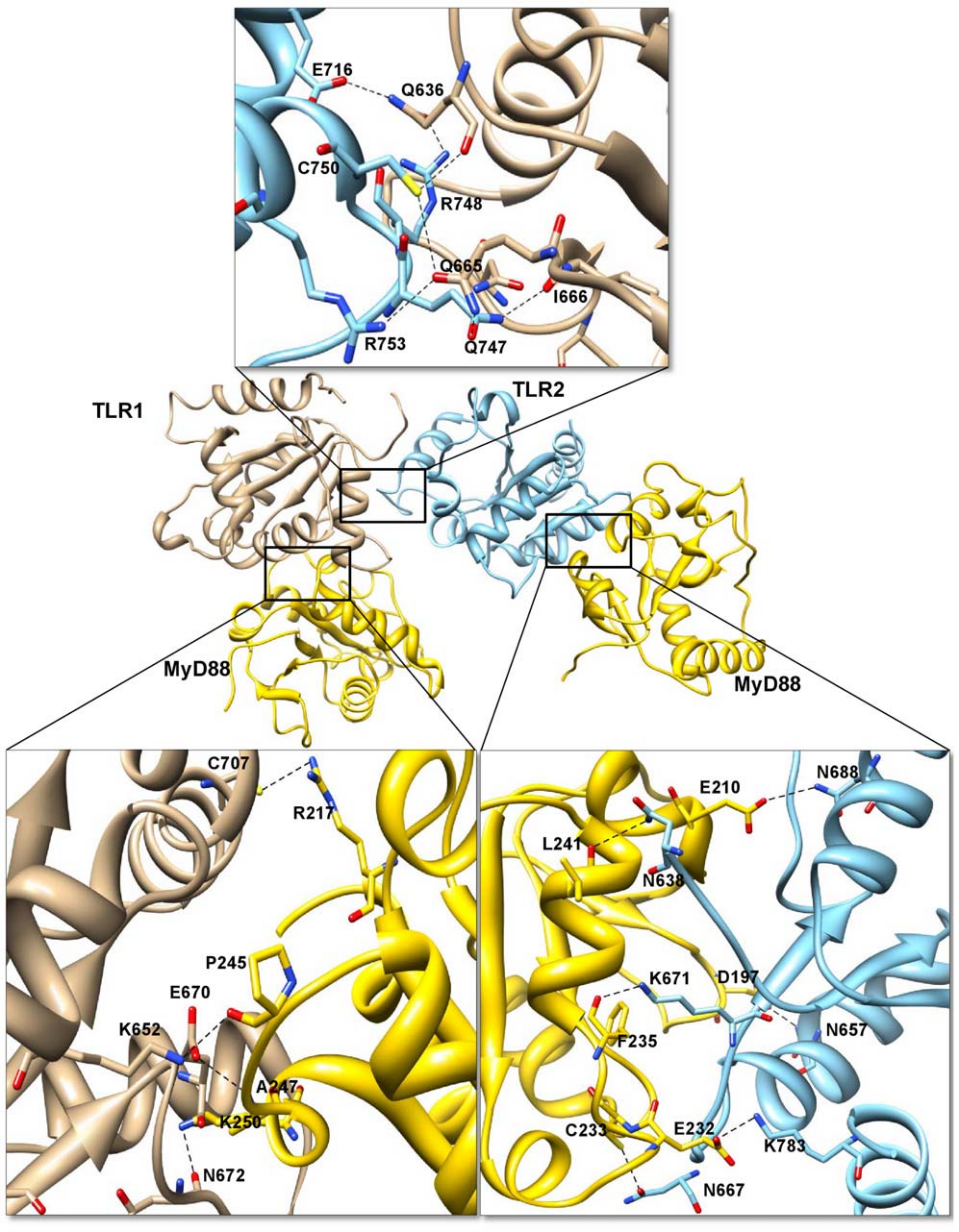
Interactions in the TLR2/1-MyD88 tetramer interface region. The TIR domains of TLR1, 2 and MyD88 are represented as ribbon models. TLR2 and 1 receptor chains A and B are shown in sky blue and tan colors, respectively. MyD88 (C and D) adapter chains are shown in gold color. The important structural motifs that contribute to the TLR1-MyD88 interface include BB-loop and αC-helix of TLR1 and CD loop, βD and αE-helix of MyD88. At the TLR2-MyD88 interface, the important structural motifs include BB-loop and αC-helix of TLR2 and beginning of BB-loop and αB-helix of MyD88. The interacting residues in the tetramer residual interface region are highlighted in boxes. Side chains of the amino acids contributing to hydrogen bond formation are represented as a stick model with the residue names and numbers shown next to them. Black dotted lines represent the hydrogen bonds.

The buried surface area at the interface of the TLR1-MyD88 complex constitutes 660 Å^2^ from the TLR1 and 769 Å^2^ from MyD88 molecule C. Similarly, the interface area of the TLR2-MyD88 complex constitutes 988 Å^2^ from TLR2 and 903 Å^2^ from MyD88 molecule D. Twenty-six residues from TLR2 chain B make contact with 28 residues from MyD88 chain D. Similarly, 27 residues from TLR1 chain A make contact with 21 residues from MyD88 chain C. Major contributions to the TLR2-MyD88 interface are made by the BB loop residues and αC-helix of TLR2 and by the beginning of the BB loop and αB-helix residues of MyD88. At the TLR1-MyD88 interface, major contributions are made by the BB loop and αC-helix residues of TLR1 and by the CD loop, βD and αE-helix residues of MyD88. A large network of hydrogen bonds and salt bridges present between the TLR1-MyD88 and TLR2-MyD88 complexes mediates this TLR2/1-MyD88 interface (Figure 7). Six hydrogen bonds are present at the interface of the TLR2-MyD88 complex and four hydrogen bonds at the interface of the TLR1-MyD88 complex, as shown in Figure S3A. Electrostatic and hydrophobic interactions are also present in both components of the complex. Two salt bridges are formed between TLR2 K783 and MyD88 E232 and between TLR2 K695 and MyD88 E210 in the TLR2-MyD88 complex. Residue K783 of TLR2 also plays a potent role in hydrogen bond formation, further stabilizing the docked complex. Similarly, two salt bridges are formed between TLR1 H708 and MyD88 P296 and between TLR1 E705 and MyD88 R251 in the TLR1-MyD88 complex. Therefore, our final docked TLR2/1-MyD88 complex is in agreement with the possibility offered by Brown et al. [45].

#### TLR2/6-MyD88 tetramer

TLR1 and 6 TIR domains share 93.8% sequence similarity and 86.8% sequence identity. Since both of these TIR domains share high sequence identity and similarity, we hypothesized that the TLR2/6-MyD88 complex could be similar to the TLR2/1-MyD88 complex. Hence, from the docking solutions, we focused on the TLR2-MyD88 complex, which has the same orientation as the TLR2/1-MyD88 complex. Previous mutagenesis studies showed that the co-varying amino acids F637, H638, N672 and P675 in TLR1 are functionally linked to MyD88 binding [45]. Therefore, using sequence alignment, we found that the corresponding residues F637, H638, N672 and P675 in TLR1 were identical to F642, H643, N677 and P680 residues in TLR6. Thus, we performed restrained docking by forcing these residues in TLR6 to bind with MyD88 and obtained the TLR6-MyD88 complex. Consequently, we superimposed the TLR2-MyD88 and TLR6-MyD88 complexes and obtained the final TLR2/6-MyD88 tetramer complex.

**Figure 8:**
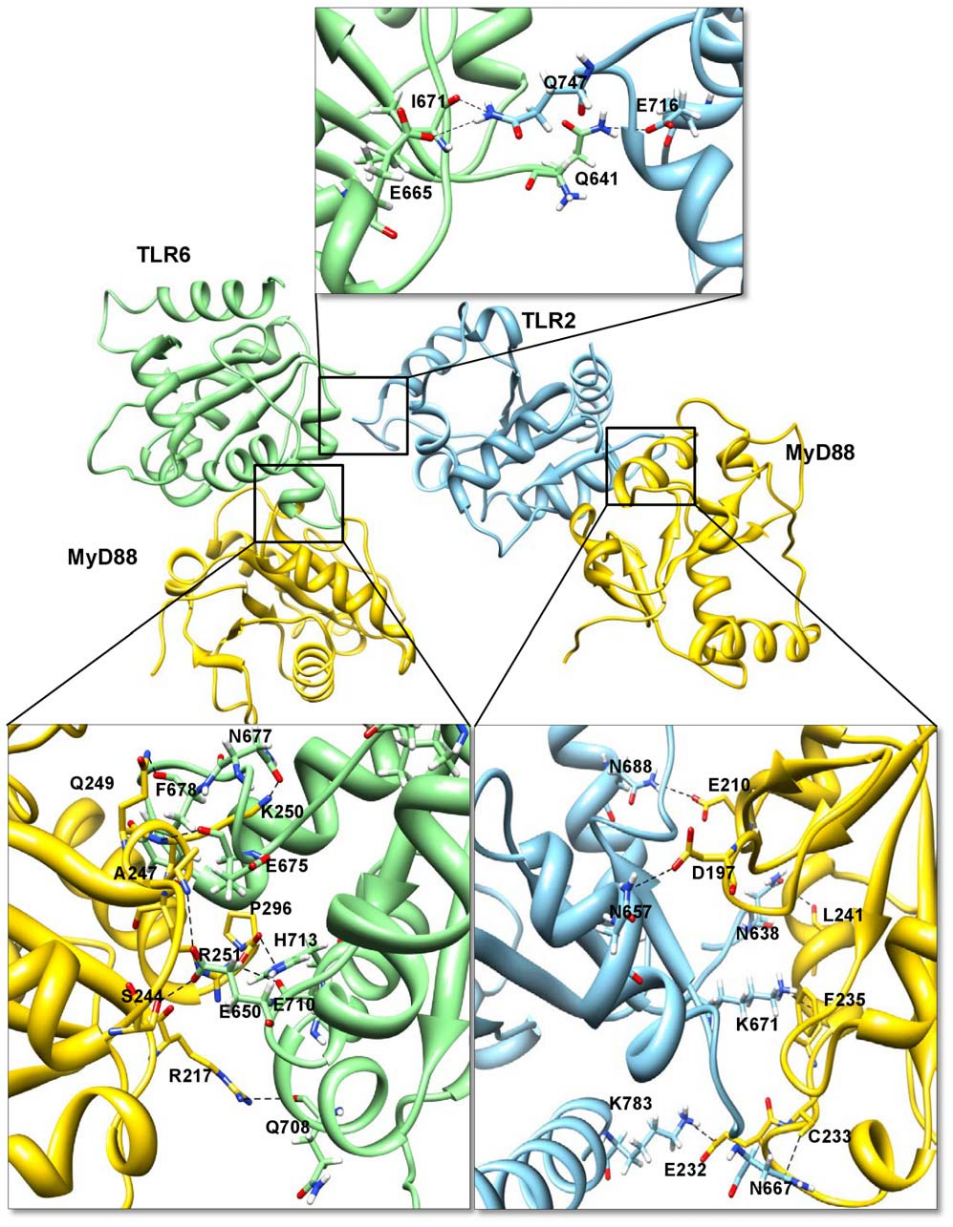
Interactions in the TLR2/6-MyD88 tetramer interface region. The TIR domains of TLR2, 6 and MyD88 are represented as ribbon models. TLR2 and 6 receptor chains A and B are shown in sky blue and light green colors, respectively. MyD88 (C and D) adapter chains are shown in gold color. Major structural motifs that contribute to the TLR6-MyD88 interface include BB-loop and αC-helix of TLR6 and CD loop, βD and αE-helix of MyD88. At the TLR2-MyD88 interface, the important structural motifs include BB-loop and αC-helix of TLR2 and beginning of BB-loop and αB-helix of MyD88. The interacting residues in the tetramer residual interface region are highlighted in boxes. Side chains of the amino acids contributing to hydrogen bond formation are represented as a stick model with the residue names and numbers shown next to them. Black dotted lines represent the hydrogen bonds.

The buried surface area at the interface of the TLR6-MyD88 complex constitutes 745 Å^2^ from the TLR6 and 814 Å^2^ from MyD88 molecule C. Similarly, the interface area of the TLR2-MyD88 complex constitutes 1024 Å^2^ from TLR2 and 941 Å^2^ from MyD88 molecule D. The TLR2-MyD88 interface residues are the same as those in the TLR2/1-MyD88 complex. Twenty-five residues from TLR6 chain A make contact with 24 residues from MyD88 chain C. Major contributions to the TLR6-MyD88 interface are made by the BB loop and αC-helix residues of TLR6 and by the CD loop, βD and αE-helix residues of MyD88 (Figure 8). Seven hydrogen bonds are present at the interface of the TLR6-MyD88 complex, as shown in Figure S3B. E650 of TLR6 forms two hydrogen bonds with residues S244 and A247 of MyD88. Strong electrostatic and hydrophobic interactions are also present in the complex. Two salt bridges are formed between TLR6 H713 and MyD88 P296 and between TLR6 E710 and MyD88 R251 in the TLR6-MyD88 complex. Residues H713 and E710 of TLR6 form both hydrogen bonds and salt bridges, further stabilizing the complex.

### Docking Studies of Inhibitory Complexes

ST2L seems to exert a negative regulatory function on TLR2 and 4-mediated NF-κB activation through sequestration of the TLR proximal signaling adapter proteins MyD88 and Mal. Furthermore, a mutant ST2L, in which Pro in box 2 (BB loop residue) of the TIR domain is mutated to His, lacks suppressive activity and is impaired in its ability to interact with the two adapter proteins [20].

#### ST2L-Mal

**Figure 9:**
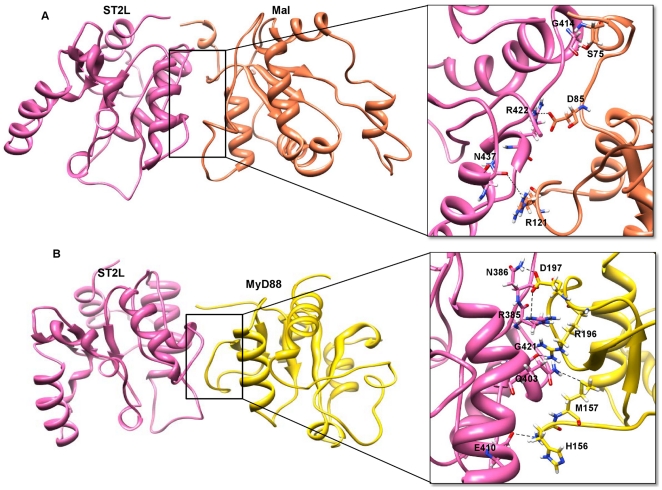
Interactions in the inhibitory interface region. (A) The TIR domains of ST2L and Mal are represented as ribbon models. ST2L and Mal chains are shown in hot pink and coral colors, respectively. The important structural motifs that contribute to the ST2L-Mal inhibitory interface include BB-loop and the beginning of βA and βB of Mal and AB and BB loops of ST2L. (B) The TIR domains of ST2L and MyD88 are represented as ribbon models. ST2L and MyD88 chains are shown in hot pink and gold colors, respectively. The important structural motifs that contribute to the ST2L-MyD88 inhibitory interface include BB-loops of both MyD88 and ST2L. Other structural motifs present at the interface area include AA-loop and αA-helix of ST2L and the beginning of αA-helix of MyD88. The interacting residues in the inhibitory residual interface region are highlighted in boxes. Side chains of the amino acids contributing to hydrogen bond formation are represented as a stick model with the residue names and numbers shown next to them. Black dotted lines represent the hydrogen bonds.

ST2L heterodimerizes with Mal, preventing interaction of Mal with the receptor TIR domain and MyD88. The ST2L-Mal docked complex has a buried surface interaction area of 770 Å^2^. Twenty-one residues from Mal and 25 residues from ST2L are present at the interface region of the docked inhibitory complex (Figure 9A). The Mal BB-loop together with the beginning of the βA and βB residues interact with the ST2L AB and BB loops, respectively. The binding mode of the ST2L-Mal complex is similar to the dimeric crystal structure of human TLR10 [43], in which the BB-loop is a main component of the interactions. The inhibitory complex is further stabilized by the formation of hydrogen bonds and a salt bridge. Three hydrogen bonds are present at the heterodimer interface (Figure S4A). Residues G414 and N437 of ST2L form hydrogen bonds with residues S75 and R121 of Mal, respectively. Furthermore, Mal D85 forms both a hydrogen bond and salt bridge with ST2L R422. Most importantly, the BB loop residues of ST2L interact with Mal critical residues Y86 (critical for MyD88 interaction) and P125 (critical for receptor interaction) at the interface of the inhibitory docked complex, preventing binding of MyD88 and the receptor TIR domain with Mal.

#### ST2L-MyD88

ST2L heterodimerizes with MyD88 in a similar manner as does the ST2L-Mal inhibitory complex. This docked inhibitory complex has a buried surface interaction area of 663 Å^2^. Seventeen residues from MyD88 and 20 residues from ST2L are present at the interface region of the docked inhibitory complex (Figure 9B). In this inhibitory complex, major interactions occur between the BB loops of both ST2L and MyD88. Other regions present at the interface area include the AA loop and αA-helix residues of ST2L and the beginning of the αA-helix residues of MyD88. It is interesting to note that the BB loop residues of ST2L interact with MyD88 critical residues R196 and D197 (critical for Mal interaction) at the interface of the inhibitory docked complex, preventing binding of Mal with MyD88. The inhibitory complex is further stabilized by the formation of hydrogen bonds and salt bridges. Five hydrogen bonds are present at the heterodimer interface (Figure S4B). Residues Q403, E410 and G421 of ST2L form hydrogen bonds with residues M157, H156 and R196 of MyD88, respectively. Furthermore, MyD88 D197 forms a double hydrogen bond with residues R385 and N386 of ST2L. Two strong salt bridges are formed between ST2L R385 and MyD88 D197 and between ST2L E410 and MyD88 H156.

Moreover, electrostatic potential studies on both inhibitory complexes revealed that the basic patches present in the adapter molecules (MyD88 and Mal - Figure S5B and D, highlighted in blue dotted circles) interact with the negatively charged surface of an inhibitor ST2L molecule (Figure S5A and C, highlighted in blue dotted circles), thereby suggesting that the predominant interactions are based on electrostatic interactions.

## Discussion

To date, the structures of the TIR domains of human TLR1 [41], TLR2 [50], TLR10 [43], IL-1RAPL [53], MyD88 [34], bacteria *Paracoccus denitrificans* [54] and plant *Arabidopsis thaliana* [55] have been determined. The TIR domains of the signaling receptors and adapters mediate the initial events that occur after activation of TLR ECDs by pathogen recognition and thus represent a focal point for the regulation of TLR signaling pathways. The hetero-oligomerization of the TIR domains of the receptor and adapter brings about the activation of the transcription factor NF-κB, which regulates the synthesis of pro-inflammatory cytokines. Most TIR domain structures solved by X-ray crystallography or NMR are monomeric and do not provide insights into the arrangement adopted by TIRs in the activated receptor. However, recent studies have revealed the dimer crystal structures of the TIR domain of human TLR10 and bacterial TIR domain. The crystal structure of the human TLR10 TIR domain was revealed to be a homodimer, the formation of which is mediated by residues from the BB-loop and αC-helix [43]. On the other hand, the bacterial TIR domain structure shows a unique dimerization interface involving the DD-loop and EE-loop residues, leaving the BB-loop highly exposed [54].

Previous studies have indicated the importance of three short sequence motifs called box 1–3 motifs, (F/Y)DA, RDXXPG and FW, which are conserved between TIR domains. Of these, box 2 motif, which resides in the so-called BB loop region, has been suggested to play a potent role in TIR-TIR interactions and specificities [41], [56], [57]. Several structural and mutational studies have pointed to the BB-, DD- and EE-loop regions as mediators of the homo- or heterodimerization function of TIR domains in bacteria and mammals [41], [43], [54]. However, neither the homotypic nor heterotypic interactions between the TIR domains of receptors and adapters are understood well. To this end, we have derived a working hypothesis for TLR2 and 4 signaling and ST2L inhibition (Figures 2 and 3) using molecular modeling studies.

Dunne et al. [35] previously attempted to model the interactions of Mal and MyD88 with TLR2 and TLR4 using monomeric receptors and adapters in the modeling process. However, it was previously shown that receptor activation leads to ligand-induced dimerization [4]. Therefore, this monomer to monomer model may not fully reflect the physiological interactions. Miguel et al. [32] generated a structural model of the TLR4 TIR dimer and used molecular docking studies to probe for the potential sites of interaction between the receptor dimer and adapter molecules (Mal and TRAM). Furthermore, Gong et al. [58] generated TLR4 and 7 signaling complexes, wherein they docked the structures of the receptor homodimer to the MyD88 homodimer to create a receptor-adapter complex. However, the functional relevance of these MyD88 homomeric interactions remains obscure since the formation of a homodimer between these TIR domains has not been observed in solution. Additionally, it has been reported that isolated MyD88 TIR is monomeric in solution, although the full-length molecule is dimerized by homotypic DD interactions [34], [36].

Our human TLR4 dimer model is supported by several reports [32], [43], [56] in which the TLR4 dimer interactions are mainly mediated by BB-loops and αC-helices. This receptor TIR dimerization generates two new scaffolds with identical surface areas that can bind specifically to Mal adapter proteins. Furthermore, our docked TLR4-Mal tetramer complex generates two symmetry-related, high affinity binding sites for the second adapter molecule, MyD88 TIR with contributions from the receptor and adapter TIRs, an idea that is supported by our high resolution docking studies as shown in Figure S6. Our TLR4-Mal-MyD88 hexamer model is the first report to show binding of MyD88 to the TLR4-Mal tetramer complex by suggesting that there is a sequential assembly process that occurs for the downstream receptor-adapter interactions. First, ligands bind to the receptor ECDs, inducing the formation of M-shaped dimers. This causes the juxtamembrane sequences at the C-termini of the ECDs to come into close proximity, which promotes formation of a receptor TIR dimer in the cytoplasmic region. This receptor TIR dimer then preferentially incorporates two Mal and then two MyD88 TIR domains into the post-receptor signaling complex. In fact, our docked hexamer complex is consistent with the recently solved structure of the myddosome [59] as well as hypotheses proposed by several studies [36], [60], wherein they predicted that if each receptor TIR dimer binds to two MyD88 TIR domains, the myddosome should be able to engage with multiple activated receptor dimers. The assembly and stoichiometry of these large and transient oligomeric complexes is difficult to study *in vivo*. Thus, our hexamer model, which is in agreement with several experimental studies, provides a basis for future structural and functional studies of TLR4 receptor-adapter TIR complexes.

Previous reports [50], [51] have suggested that the DD loop side of TLR2 might interact with the BB loop region of TLR1 or 6. Based on this hypothesis, we carried out docking studies and extracted the TLR2/1 and TLR2/6 complexes. TIR heterodimer interactions are maintained mainly by the DD loop of TLR2 and BB loop regions of TLR1 or 6, which are highly conserved among different TIRs. A previous study indicated that Mal is dispensable in TLR2 signaling at high ligand concentrations, with MyD88 probably coupling to the TLR2 receptor complex at sufficient levels to allow for activation [52]. This alternate Mal-independent pathway could contribute to signaling as discussed in several studies [37], [45], [52]. In contrast to the intensively studied TLR2/1 heterodimerization, structural information about the TLR2/6 heterodimerization is lacking. Since both the TLR1 and 6 TIR domains share high sequence identity and similarity, we predicted that the TLR2/6-MyD88 complex is similar to the TLR2/1-MyD88 complex and thus proposed functionally important TLR6 TIR residues with respect to MyD88 binding. Therefore, our models of the receptor dimer (TLR2/1 and TLR2/6) docked to the MyD88 adapters provide additional information for structural interpretation.

Based on the crystal structures and mutational data, several structural models of heteromeric TIR-TIR interactions have been proposed that suggest the importance of the so-called BB loop [37], [61]. Consequently, in our current studies, we modeled the complexes of the TLR4-Mal-MyD88 hexamer and the TLR2/1-MyD88 and TLR2/6-MyD88 tetramers based on heteromeric TIR-TIR interactions, which means that they are likely more physiologically relevant. This observation suggests that MyD88 binds simultaneously and possibly cooperatively with the receptor TIR scaffold. Moreover, our TLR4-Mal-MyD88 hexamer model suggests that the initial binding of Mal to the receptor TIR domains contributes to the formation of symmetry-related sites for the binding of the second adapter molecule, MyD88. Structural insights from these studies may aid our understanding of the molecular mechanisms by which TIR domain receptors and adapters interact and participate in signaling.

Our model predictions of inhibitory docked complexes (ST2L-Mal and ST2L-MyD88) revealed that ST2L heterodimerizes with Mal and MyD88 by occupying their receptor-adapter and adapter-adapter interacting sites, thus preventing the engagement of signaling adapter proteins (Mal and MyD88) into the post-receptor signaling complex. Moreover, our final docked complexes (TLR4-Mal-MyD88 hexamer, TLR2/1-MyD88 tetramer and TLR2/6-MyD88 tetramer) showed the importance of BB-loop residues that are involved in receptor-receptor, receptor-adapter and adapter-adapter interactions. Detailed structural analysis of our final ST2L-MyD88 inhibitory complex revealed major interactions between the BB loops and αA-helices of both the ST2L and MyD88 TIR domains. Additionally, our final docked ST2L-Mal inhibitory complex revealed major interactions between the BB loops of both the ST2L and Mal TIR domains. In both the modeled inhibitory complexes, the BB-loop of ST2L plays a key role in binding, which is in agreement with the suggestions of previous studies [20], [62]. All these observations highlight the strong molecular affinity of ST2L as an inhibitor.

In summary, our work depicts a residue-detailed structural framework of ST2L inhibiting the TLR2/1, TLR2/6 and TLR4 signaling pathways. Furthermore, our modeling complexes also provide structural insights into the TIR domain architecture of the TLR2 and 4 downstream signaling pathways. Our studies can be utilized to identify TIR domain surfaces that mediate functional TIR-TIR interactions as a basis of rational design of therapeutics that specifically target TLR signaling.

## Supporting Information

Figure S1 **Intermolecular H-bonding in the TLR4 receptor-adapter interface region.** (A) The residues contributing to hydrogen bond formation in the TLR4 dimer interface are shown. A and B chains represent TLR4 TIR receptors. (B) The residues contributing to hydrogen bond formation in the TLR4-Mal tetramer interface are shown. A, B chains represent TLR4 TIR receptors and C, D chains represent Mal adapters. (C) The residues contributing to hydrogen bond formation in the TLR4-Mal-MyD88 hexamer interface are shown. A, B chains represent TLR4 TIR receptors, C, D chains represent Mal adapters and E, F represent MyD88 adapters. Black dotted lines represent the hydrogen bonds.
(TIF)

Figure S2 **Surface charge distribution of the TIR domains of TLR4, Mal and MyD88.** Electrostatic surface potential representations of the TIR domains of TLR4, Mal and MyD88 with blue-colored regions indicating positively charged basic patches and red-colored regions indicating negatively charged acidic patches. (A) TLR4 dimer, (B) Mal, (C) TLR4-Mal tetramer and (D) MyD88 TIR domain models. The blue color dotted circles represent the areas which are involved in the interactions between the receptor (TLR4) and adapter (Mal and MyD88) molecules.
(TIF)

Figure S3 **Intermolecular H-bonding in the interface region of TLR2/1-MyD88 and TLR2/6-MyD88 complexes.** (A) The residues contributing to hydrogen bond formation in the TLR2/1-MyD88 tetramer interface region are shown. A and B chains represent TLR2 and TLR1 TIR receptors. (B) The residues contributing to hydrogen bond formation in the TLR2/6-MyD88 tetramer interface region are shown. A and B chains represent TLR2 and TLR6 TIR receptors. Black dotted lines represent the hydrogen bonds.
(TIF)

Figure S4 **Intermolecular H-bonding in the interface region of inhibitory complexes.** (A) The residues contributing to hydrogen bond formation in the ST2L-Mal interface region are shown. A and B chains represent ST2L and Mal TIRs. (B) The residues contributing to hydrogen bond formation in the ST2L-Mal interface region are shown. A and B chains represent ST2L and Mal TIRs. Black dotted lines represent the hydrogen bonds.
(TIF)

Figure S5 **Surface charge distribution of the TIR domains of ST2L, Mal and MyD88.** Electrostatic surface potential representations of the TIR domains of ST2L, Mal and MyD88 with blue-colored regions indicating positively charged basic patches and red-colored regions indicating negatively charged acidic patches. A and C, ST2L. B, MyD88. D, Mal TIR domain models. The blue color dotted circles represent the areas which are involved in the interactions between the inhibitor (ST2L) and adapter (Mal and MyD88) molecules.
(TIF)

Figure S6 **Symmetry-related binding sites for MyD88.** Electrostatic surface potential representations of the TIR domains of the TLR4-Mal tetramer complex showing two symmetry-related binding sites for MyD88. The two dotted arrow lines show the identical scaffolds in the TLR4-Mal tetramer complex for binding of the second adapter molecule, MyD88.
(TIF)
