# Supplementary figures and images for: In Silico Approach to Inhibition of Signaling Pathways of Toll-Like Receptors 2 and 4 by ST2L
Source: PLoS One. 2011 Aug 29;6(8):e23989. doi: 10.1371/journal.pone.0023989 (PMC3163686; doi:10.1371/journal.pone.0023989)

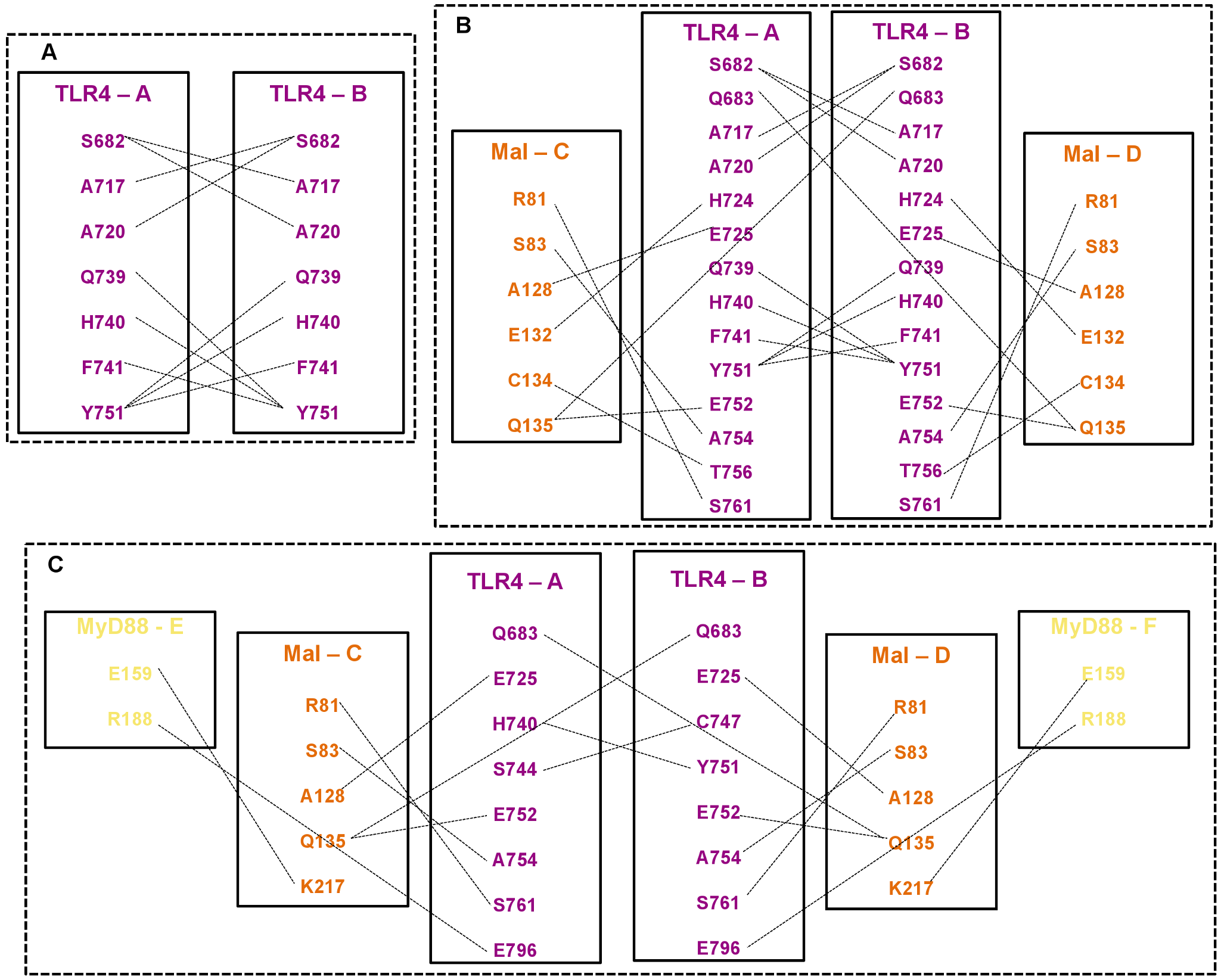

Supplement: Figure S1 — Intermolecular H-bonding in the TLR4 receptor-adapter interface region. (A) The residues contributing to hydrogen bond formation in the TLR4 dimer interface are shown. A and B chains represent TLR4 TIR receptors. (B) The residues contributing to hydrogen bond formation in the TLR4-Mal tetramer interface are shown. A, B chains represent TLR4 TIR receptors and C, D chains represent Mal adapters. (C) The residues contributing to hydrogen bond formation in the TLR4-Mal-MyD88 hexamer interface are shown. A, B chains represent TLR4 TIR receptors, C, D chains represent Mal adapters and E, F represent MyD88 adapters. Black dotted lines represent the hydrogen bonds. (TIF) [file pone.0023989.s001.tif]

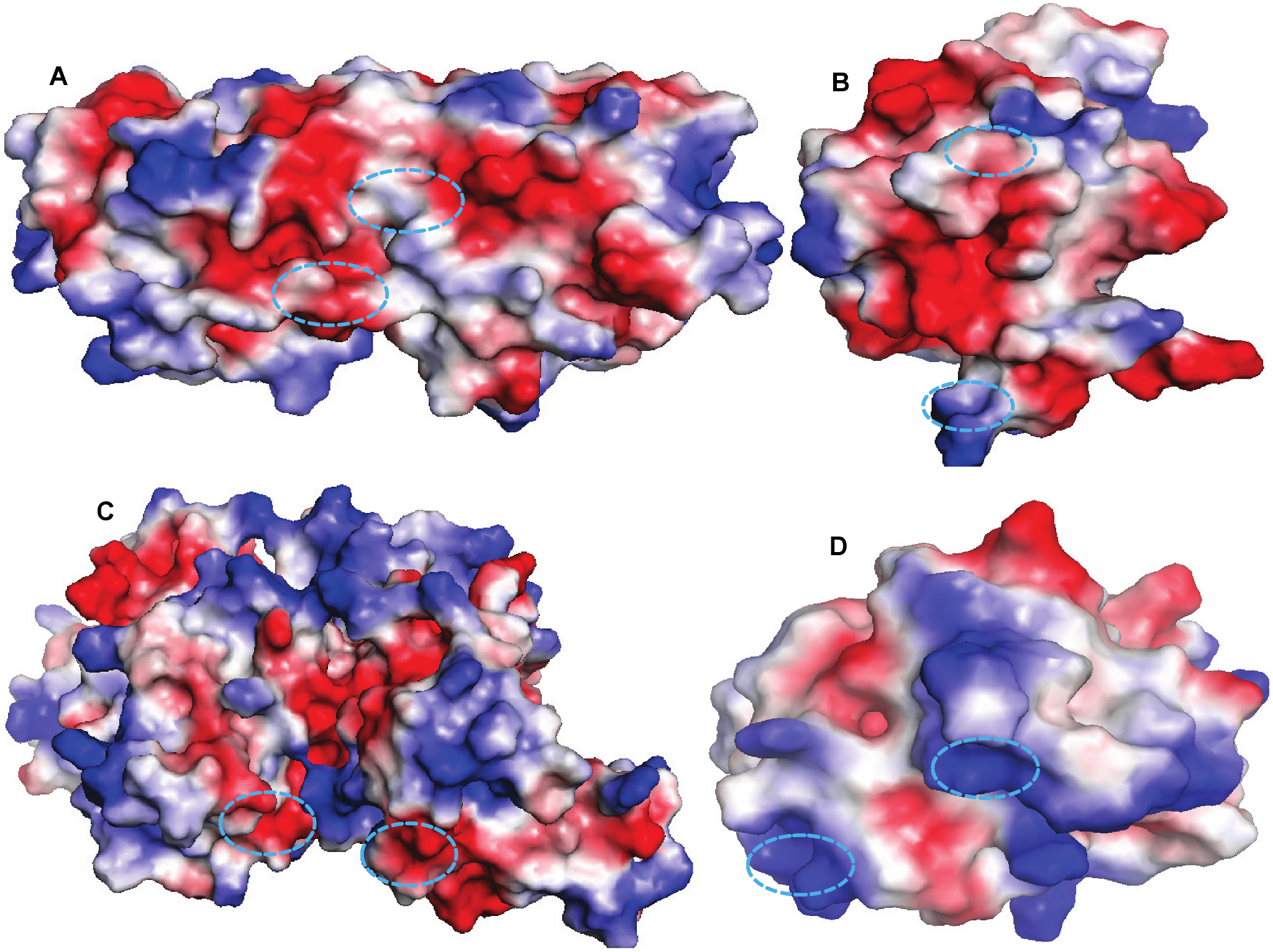

Supplement: Figure S2 — Surface charge distribution of the TIR domains of TLR4, Mal and MyD88. Electrostatic surface potential representations of the TIR domains of TLR4, Mal and MyD88 with blue-colored regions indicating positively charged basic patches and red-colored regions indicating negatively charged acidic patches. (A) TLR4 dimer, (B) Mal, (C) TLR4-Mal tetramer and (D) MyD88 TIR domain models. The blue color dotted circles represent the areas which are involved in the interactions between the receptor (TLR4) and adapter (Mal and MyD88) molecules. (TIF) [file pone.0023989.s002.tif]

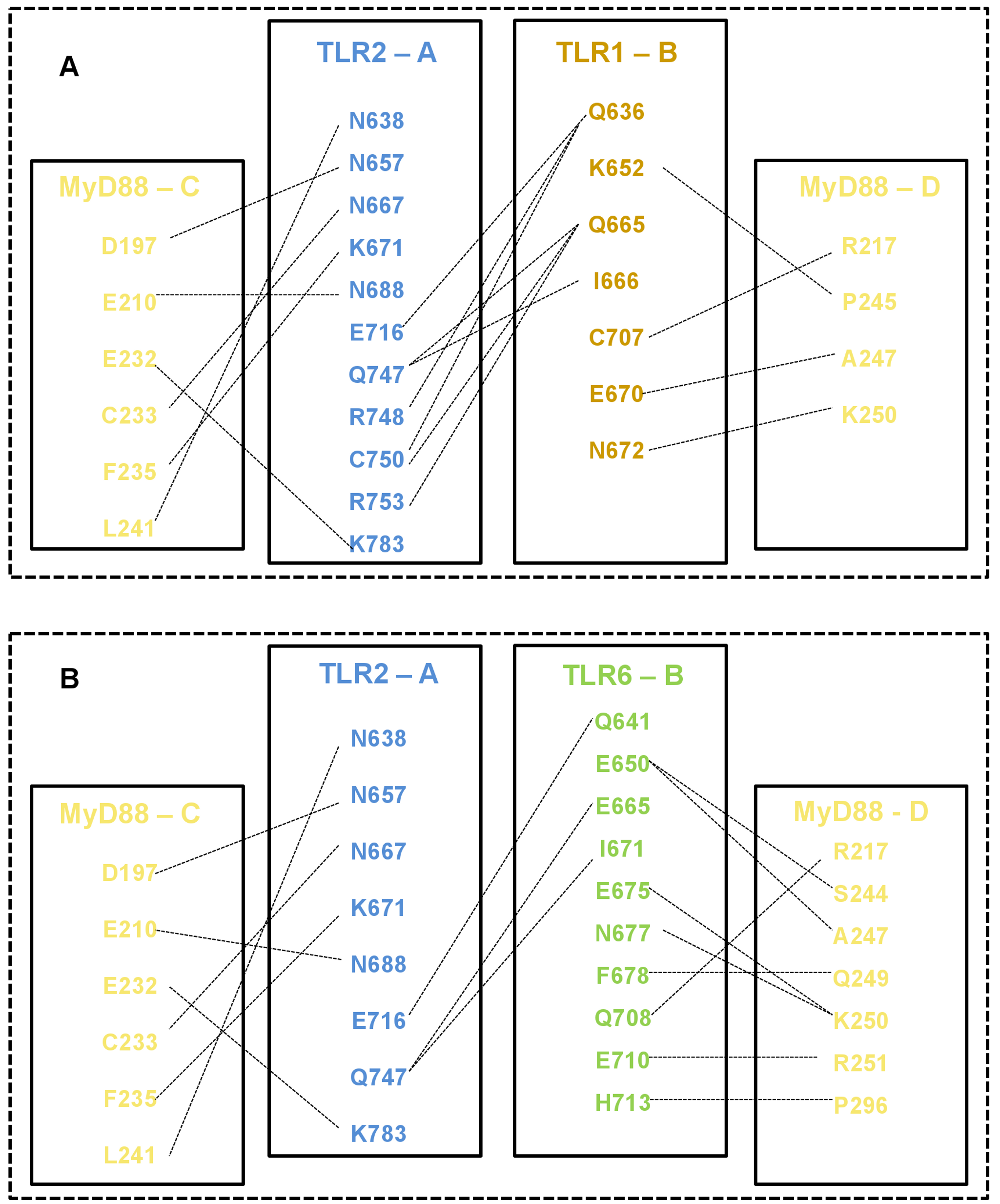

Supplement: Figure S3 — Intermolecular H-bonding in the interface region of TLR2/1-MyD88 and TLR2/6-MyD88 complexes. (A) The residues contributing to hydrogen bond formation in the TLR2/1-MyD88 tetramer interface region are shown. A and B chains represent TLR2 and TLR1 TIR receptors. (B) The residues contributing to hydrogen bond formation in the TLR2/6-MyD88 tetramer interface region are shown. A and B chains represent TLR2 and TLR6 TIR receptors. Black dotted lines represent the hydrogen bonds. (TIF) [file pone.0023989.s003.tif]

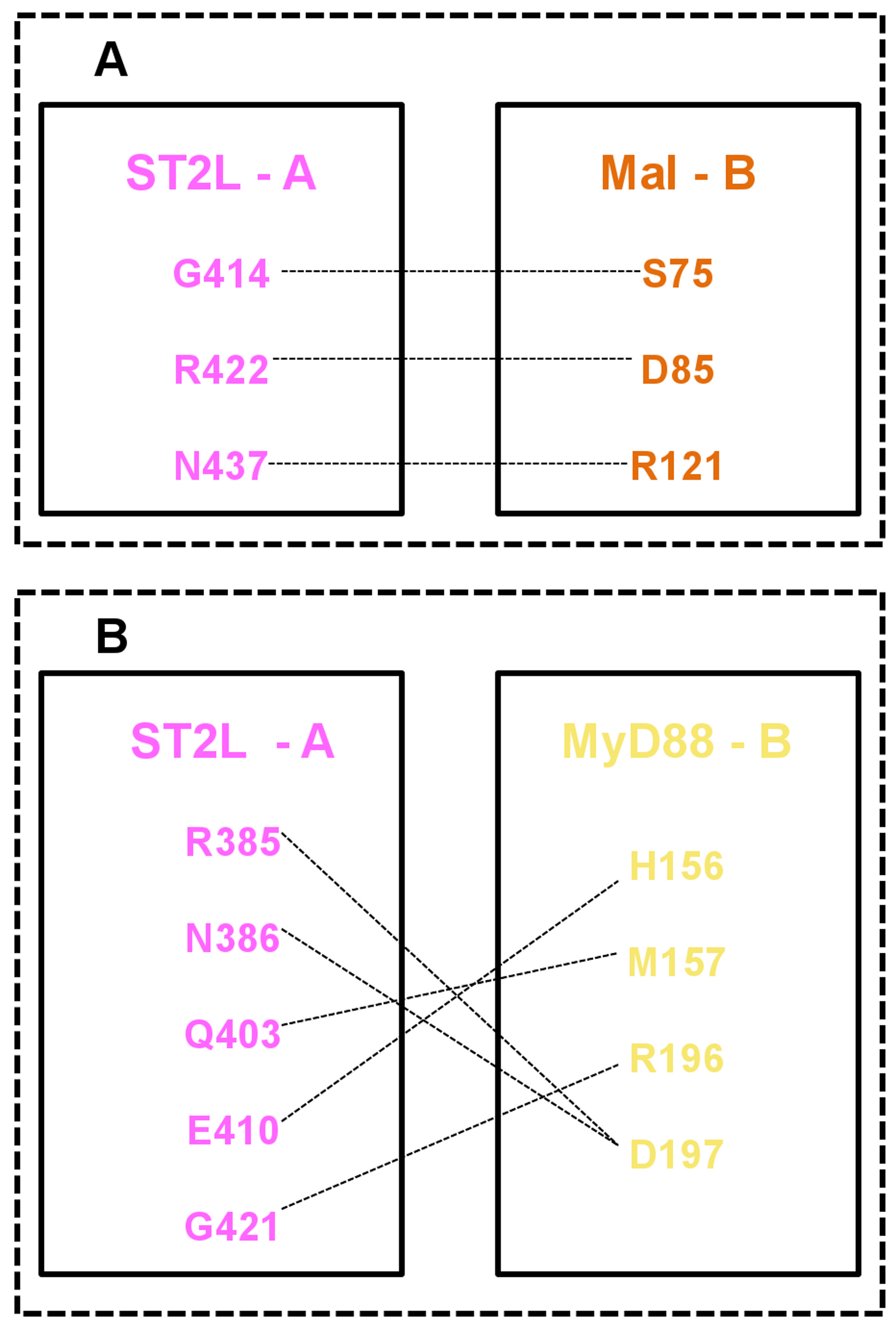

Supplement: Figure S4 — Intermolecular H-bonding in the interface region of inhibitory complexes. (A) The residues contributing to hydrogen bond formation in the ST2L-Mal interface region are shown. A and B chains represent ST2L and Mal TIRs. (B) The residues contributing to hydrogen bond formation in the ST2L-Mal interface region are shown. A and B chains represent ST2L and Mal TIRs. Black dotted lines represent the hydrogen bonds. (TIF) [file pone.0023989.s004.tif]

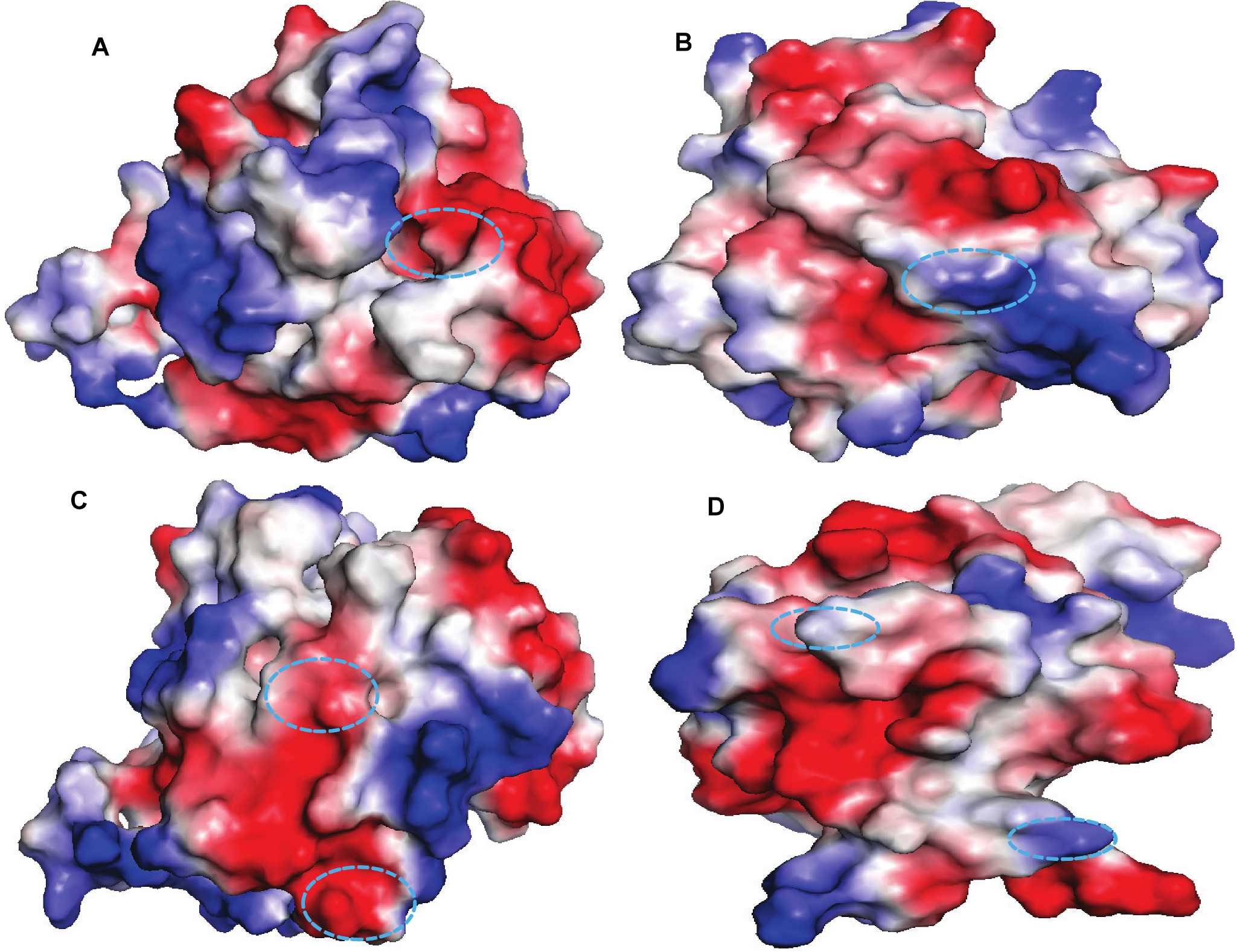

Supplement: Figure S5 — Surface charge distribution of the TIR domains of ST2L, Mal and MyD88. Electrostatic surface potential representations of the TIR domains of ST2L, Mal and MyD88 with blue-colored regions indicating positively charged basic patches and red-colored regions indicating negatively charged acidic patches. A and C, ST2L. B, MyD88. D, Mal TIR domain models. The blue color dotted circles represent the areas which are involved in the interactions between the inhibitor (ST2L) and adapter (Mal and MyD88) molecules. (TIF) [file pone.0023989.s005.tif]

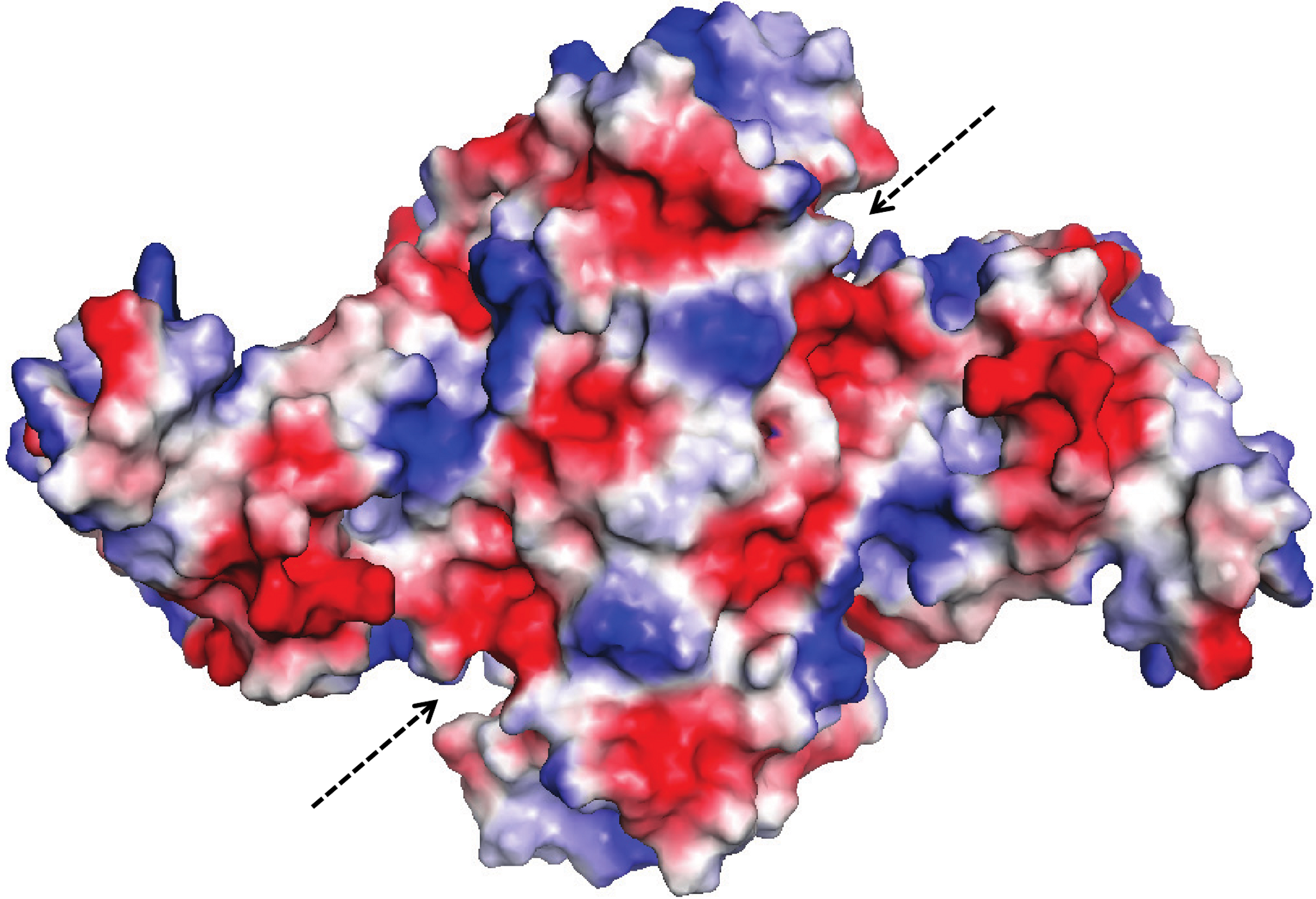

Supplement: Figure S6 — Symmetry-related binding sites for MyD88. Electrostatic surface potential representations of the TIR domains of the TLR4-Mal tetramer complex showing two symmetry-related binding sites for MyD88. The two dotted arrow lines show the identical scaffolds in the TLR4-Mal tetramer complex for binding of the second adapter molecule, MyD88. (TIF) [file pone.0023989.s006.tif]
